# Reinterpretation of an endangered taxon based on integrative taxonomy: The case of *Cynara baetica* (Compositae)

**DOI:** 10.1371/journal.pone.0207094

**Published:** 2018-11-28

**Authors:** Sergi Massó, Jordi López-Pujol, Roser Vilatersana

**Affiliations:** 1 Botanic Institute of Barcelona (IBB, CSIC-ICUB), Barcelona, Catalonia, Spain; 2 BioC-GReB, Laboratori de Botànica, Facultat de Farmàcia, Universitat de Barcelona, Barcelona, Catalonia, Spain; Universidad de Sevilla, SPAIN

## Abstract

The Strait of Gibraltar, the gateway between the Atlantic Ocean and the Mediterranean Sea, has a convulsive geological history, with recurring closing and opening events since the late Miocene. As a consequence, this region has played a major role in the evolutionary history of many species. *Cynara baetica* (Compositae) is a diploid perennial herb distributed in both sides of this strait. It is currently subdivided into two subspecies: *C*. *baetica* subsp. *baetica* for the Spanish populations, and *C*. *baetica* subsp. *maroccana* for the Moroccan ones. Following three different approximations of species delimitation, including phylogenetic and population genetic analyses (based on three AFLP primer combinations and two intergenic spacers of cpDNA), ecological niche modeling (ENM) and morphological studies, this taxon is investigated and reinterpreted. The results obtained showed a clear genetic, morphological and ecological differentiation between the two taxa and the important role played by the Strait of Gibraltar as a geographical barrier. Based on this evidence, the current taxonomic treatment is modified (both taxa should recover their specific rank) and specific conservation guidelines are proposed for the newly delimited taxa.

## Introduction

The Strait of Gibraltar (SG) is the gateway between the Atlantic Ocean and the Mediterranean Sea and separates the Eurasian and African plates at their westernmost parts. It is a region with a convulsive geological history, and the strait has experienced recurring closing and opening events since the late Miocene (ca. 8 Mya [1]); during these events, SG acted both as a corridor and barrier to biodiversity. At the end of this period, during the Messinian salinity crisis (ca. 6–5.3 Mya [1]), when the sea level was approximately 120–150 m lower than at present [2] and islands between Africa and Europe appeared [3], SG was frequently used as a land bridge for the migration and expansion of species [4,5]. The last re-opening of the strait (5.3 Mya [6]) triggered independent evolutionary processes on both sides [7]. During the Mid-Pliocene (ca. 3.6 Mya), the climatic conditions of the region changed resulting in a Mediterranean climate with strong oceanic influence [8] and ever since, these remained relatively stable, despite the fact that there were major climatic fluctuations of the Pleistocene (2.6 Mya) [9]. During the cold periods of the Pleistocene, the Mediterranean Basin acted as a refugium for many northern European plant species [10], and the distance across the strait became reduced to ca. 10 km owing to a drop in sea level [11]. In fact, Miocene, Pliocene and Pleistocene events (e.g., tectonic movements, onset of Mediterranean climate, and ice ages) are the main factors causing the current distribution and population genetic structure of many Mediterranean plants [12].

Owing to its particular climate and geology, the SG region can be considered an important area of remarkable biogeographical and ecological interest. According to Médail and Quézel [13] is part of one of the most important ten hot-spots of plant biodiversity within the Mediterranean Basin, namely the Baetic-Rifan complex. This region contains many late Paleogene-Neogene relicts as well as narrow endemic species of recent origin [14], and accounts for some 18% of the Mediterranean Basin’s plant biodiversity [15]. Floristic similarities between both sides of SG have been extensively studied during the last decades (e.g., [15–17]). In fact, the Mediterranean flora in this region comprises ca. 5000 species [18] and ca. 75% of these occur both on the Iberian Peninsula and in northern Morocco [16].

Studies of plant and animal species occurring in the SG region are common nowadays, often showing ambiguous results regarding their genetic patterns. On one hand, strong genetic differentiation (leading thus to independent lineages) has been detected in some plant species (e.g., *Carex helodes* Link [18], *Saxifraga globulifera* Desf. [19]) including complete cross-strait differentiation (see Rodríguez-Sánchez et al. [9], and references therein) suggesting that the Strait acted as a strong geographic barrier, only allowing migration and gene flow in certain lineages, and producing allopatric speciation in others. On the other hand, genetic cohesion (due to active gene flow) between both sides has also been reported (e.g., *Hypochaeris salzmanniana* DC. [20]; *Calicotome villosa* (Poir.) Link [21]) suggesting that SG may also have provided a route for plant migration or, at least, did not present a major barrier to that process.

*Cynara* L. is a Mediterranean genus of the family Compositae and, currently, includes nine species [22]. According to the fossil-calibrated phylogeny of the tribe Cardueae by Barres et al. [23], speciation events in the genus *Cynara* date back to about 12 Mya, before the Messinian salinity crisis. Three species are distributed throughout southern Europe and northern Africa: *C*. *cardunculus* L., *C*. *humilis* L. and *C*. *baetica* (Spreng.) Pau, the last being restricted to southern Spain and northern Morocco. *Cynara baetica* is a diploid (2*n* = 34 [24]) perennial herb, characterized by dark margins to the capitulum bracts and whitish veins on the lower surface of the leaves. Its flowering period is from July to September. As in the remaining *Cynara* species, *C*. *baetica* is self-compatible but predominantly cross-pollinated [25]. Although no specific studies have been undertaken for *C*. *baetica*, based on other *Cynara* species (e.g., [26,27]), these are likely to be bees (*Apis mellifera*) and bumblebees (*Bombus* sp.).

The most recent taxonomic treatment [28] divides *C*. *baetica* into two subspecies: *C*. *baetica* subsp. *baetica* and *C*. *baetica* subsp. *maroccana* Wikl. Both taxa grow in open areas on calcareous bedrocks in deep and nitrogenous soils but at different altitudes. *Cynara baetica* subsp. *baetica* occurs between 500 and 1700 m, and is cited within and around the Baetic Ranges, in southern Spain; meanwhile *C*. *baetica* subsp. *maroccana* grows at somewhat higher altitudes (from 900 to 2100 m) in central and northern Morocco (Rif and Atlas mountain ranges). Whereas subsp. *baetica* is listed as “Vulnerable” (VU) in Spain [29], there is no protection or red list in Morocco. As in other species of the genus, *C*. *baetica* is prone to hybridization and introgression [30]; in northern Morocco, for example, some wild hybrids between *C*. *baetica* and *C*. *humilis* have been described [28,31]. However, manual cross-pollination of *C*. *cardunculus* and *C*. *baetica* produce only very few seeds, and the F1 hybrids are generally sterile [25].

Over time, the taxonomical treatment of *C*. *baetica* has changed several times. This taxon was first recorded in the Iberian Peninsula by Lagasca [32] and named *Cirsium horridum*, but later assigned to *Cynara alba* by de Candolle [33]. Meanwhile, Ball [34] described *Cynara hystrix* from Morocco. More than a century later, Wiklund [28] merged *C*. *alba* and *C*. *hystrix* to form a single species (*C*. *baetica*), although she recognized them as seperate subspecies based on the morphology of their petals and bracts (see Table 1); Iberian and Moroccan populations were eventually assigned to *C*. *baetica* subsp. *baetica* and *C*. *baetica* subsp. *maroccana*, respectively.

**Table 1:** Morphological differences between the two described subspecies of *Cynara baetica* based on Wiklund’s [28] study.

.	*C*. *baetica* subsp. baetica	*C*. *baetica* subsp. *maroccana*
**Florets color**	Whitish	Bright lilac
**Involucral bracts**		
Color	Pale green	Pale green, tinged with purple
Protrusion length	17–30 mm	(20–)28–32 mm
Spines length	2.5–6 mm	5–10 mm

Accurate identification at species level is an essential prerequisite of biological investigations, whatever their purpose. But alongside this, scientists have long debated the concept of what defines a species. This constantly changes in the light of new data presented by evolutionary biologists, and many of these data sets are often incompatible with each other (summarized in de Queiroz [35]). Recently, however, an emerging consensus has proposed that species should be delimited as evolutionarily distinct lineages based on results obtained by means of a range of techniques used in conjunction, including genetic, morphological, and ecological niche studies, as well as other lines of evidence [36–38]. This multidisciplinary approach to define species limits is more difficult to apply to plants than to animals due to intralineage phenotypic variation and frequent interlineage introgressions in the former [39]. This is especially true for taxa occurring in poorly studied regions with lack of specimens, where defining species objectively and consistently has a higher degree of difficulty. Accurate identification is also essential in conservation, since erroneous species boundaries can lead to the incorrect use of strategies for conservation purposes [40].

The aims of the present study are to: (i) analyze the genetic diversity within and among *C*. *baetica* populations; (ii) infer the phylogeographic pattern of *C*. *baetica* and the role of the Strait of Gibraltar as a barrier; (iii) evaluate the morphological, genetic and ecological differences between both subspecies; and (iv) provide guidelines on their conservation. In order to achieve these goals, three different approaches were employed for species delineation. These were, population genetic analyses, nuclear and plastid DNA markers in conjunction with ecological niche modeling (ENM), and morphological studies. These three different techniques have proved to be reliable in the phylogenetic and phylogeographical study of populations, as recently demonstrated in the literature (e.g., Jones et al. [41] and Aleksić et al. [42]).

## Material and methods

### Ethics statements

Due to the conservation and legal status of this species in Spain, the Spanish localities were collected thanks to the collection permission issued by Consejería de Medio Ambiente de la Junta de Andalucía (resolution of 26/08/2011).

### Genetic analyses

#### Sampling

**Fig 1:**
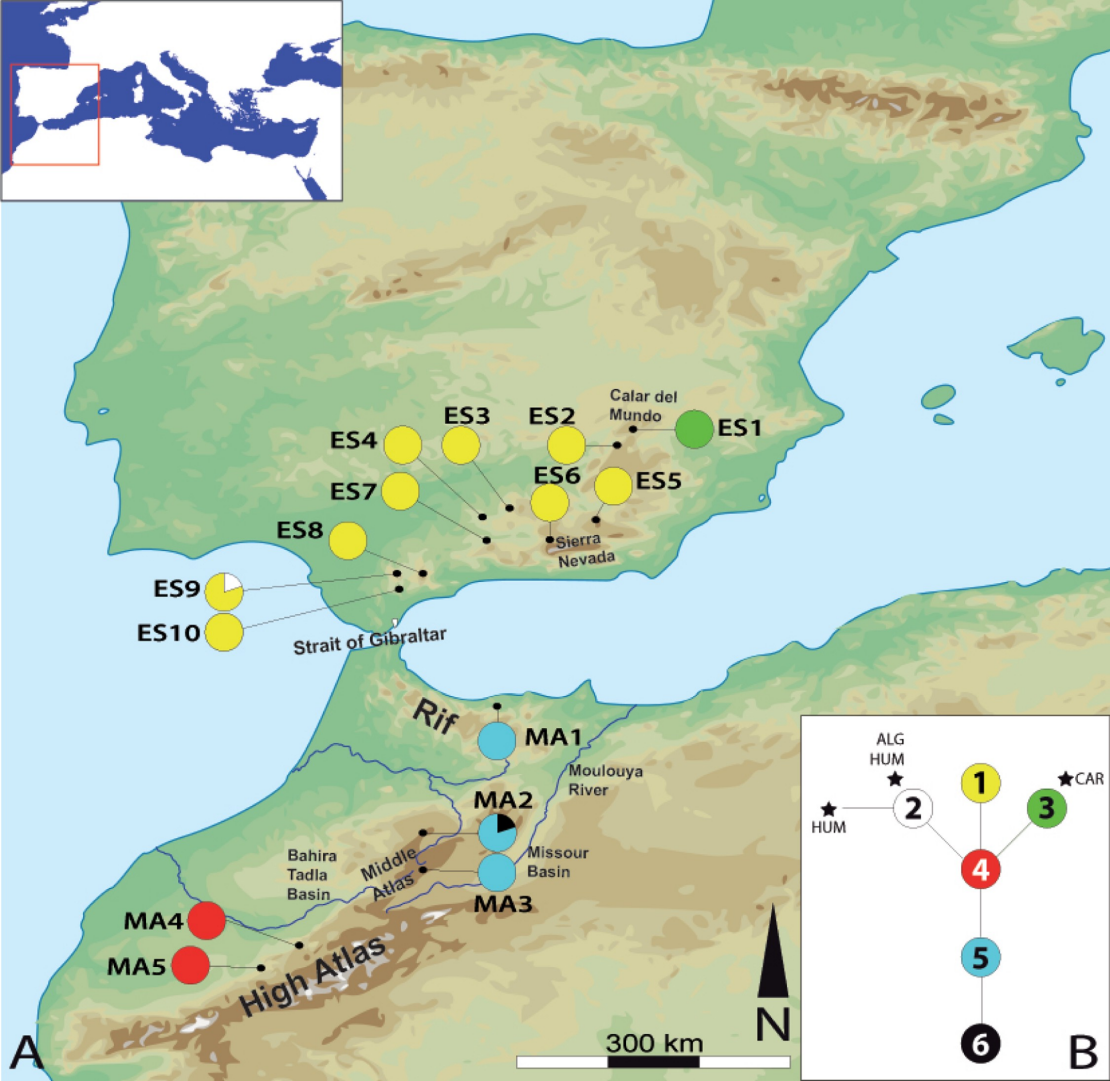
Location and haplotypes of the studied Cynara baetica populations. (A) Location and haplotype distribution of the 15 studied populations of *Cynara baetica*. (B) Statistical parsimony network of *C*. *baetica* haplotypes (indicated by numbers). Stars indicate three outgroups (ALG = *C*. *algarbiensis*; CAR = *C*. *cardunculus*; HUM = *C*. *humilis*). All the geographical features referred to in the text are also indicated.

Fifteen populations were sampled, covering the entire distribution range of *C*. *baetica*, 10 from the Iberian Peninsula and five from Morocco (Fig 1). When possible, leaf material from 10 plants per population was collected and stored in silica gel (Table 2). Voucher specimens were deposited at the Herbarium of the Botanical Institute of Barcelona (BC). Individuals of three *Cynara* species were included in the study as outgroups: *C*. *algarbiensis* Cosson ex Mariz, *C*. *humilis* and *C*. *cardunculus*. Information about all the material used in genetic analyses is detailed in Table 2.

**Table 2:** Population dataset used in this study. Code of *Cynara baetica* populations or outgroup species, locations, coordinates, altitude (Alt.), number of samples used in plastid DNA study (*N*_cp_), number of haplotypes detected (Hap.), number of samples used in AFLP study (*N*_AFLP_), number of private (Private) and fixed (Fixed; in parentheses) markers, percentage of polymorphic loci (*PLP*) and number of polymorphic loci (NL), average gene diversity (*H*j), band richness after rarefaction (*B*r_5_), and index of association modified to remove the dependency of sample size (*r*_d_) (* = p < 0.001; ns = not significant).

Code	Location	Coordinates	Alt. (m)	*N*_cp_	Hap.	*N*_AFLP_	Private (Fixed)	*PLP* (NL)	*H*j (± SE)	*B*r(5)	*r*_*d*_
**IBERIAN PENINSULA**											
ES1	Spain: Albacete, Calar del Mundo	38°29’29” N 02°18’47” W	1000	5	3	8	5 (1)	20.9 (44)	0.130 ± 0.011	1.182	0.052*
ES2	Spain: Jaén, Hornos	38°13’57” N 02°39’48” W	1260	5	1	10	0 (0)	16.6 (35)	0.089 ± 0.010	1.223	0.081*
ES3	Spain: Jaén, Locubín	37°33’58” N 03°52’18” W	1100	5	1	9	0 (0)	21.3 (45)	0.117 ± 0.009	1.206	0.052*
ES4	Spain: Córdoba, Los Villares	37°24’08” N 04°17’24” W	680	5	1	5	0 (0)	12.3 (26)	0.098 ± 0.010	1.128	0.147*
ES5	Spain: Granada, Gor	37°22’10” N 02°58’10” W	1190	5	1	10	1 (0)	22.3 (47)	0.126 ± 0.011	1.245	0.046*
ES6	Spain: Granada, El Purche	37°08’11” N 03°28’23” W	1500	5	1	10	1 (0)	19.0 (40)	0.098 ± 0.010	1.219	0.019^ns^
ES7	Spain: Málaga, Alfarnate	36°59’39” N 04°15’37” W	1000	5	1	9	1 (0)	68.2 (144)	0.142 ± 0.010	1.209	0.052*
ES8	Spain: Málaga, Puerto Martínez	36°48’13” N 04°51’20” W	730	5	1	10	0 (0)	14.7 (31)	0.103 ± 0.010	1.223	0.099*
ES9	Spain: Cádiz, Puerto Boyar	36°45’16” N 05°23’39” W	1100	5	1, 2	8	0 (0)	22.7 (48)	0.132 ± 0.010	1.188	0.120*
ES10	Spain: Málaga, Cortes	36°37’02” N 05°20’32” W	610	5	1	9	3 (0)	16.6 (35)	0.094 ± 0.009	1.227	0.058*
**Population mean**							**1 (0.1)**	**23.5 (49.5)**	**0.113** ± 0.009		**0.072**
**Subspecies level**				**50**	**1, 2, 3**	**88**	**45 (5)**	**43.6 (92)**	**0.163 ± 0.011**		0.028*
**MOROCCO**											
MA1	Morocco: Al Hoceïma, Bni Hadifa	35°00’34” N 04°11’22” W	1200	5	5	10	2 (1)	55.5 (117)	0.131 ± 0.011	1.184	0.028*
MA2	Morocco: Ifrane, Ifrane	33°30’16” N 05°01’24” W	1800	5	5	8	2 (0)	71.1 (150)	0.202 ± 0.011	1.186	0.056*
MA3	Morocco: Ifrane, Col du Zad	33°02’27” N 05°03’30” W	2100	5	5, 6	9	0 (0)	60.7 (128)	0.151 ± 0.010	1.182	0.092*
MA4	Morocco: Azilal, Afourer	32°10’57” N 06°31’16” W	1100	5	4	10	0 (0)	12.8 (27)	0.058 ± 0.008	1.195	0.111*
MA5	Morocco: Azilal, Demnate	31°45’55” N 07°00’45” W	900	5	4	10	3 (2)	12.8 (27)	0.077 ± 0.008	1.205	0.083*
**Mean**							**0.8 (0.6)**	**42.6 (89.8)**	**0.128 ± 0.010**		**0.074**
**Subspecies level**				**25**	**4, 5, 6**	**47**	**20 (1)**	**67.3 (142)**	**0.195 ± 0.012**		0.080*
**Outgroups**											
*C*.*algarbiensis*	Portugal: Algarve, Manchique	37°18’32” N 08°36’27” W	780	2	2	—	—	—	—	—	—
*C*. *cardunculus*-1	Portugal: Algarve, Moncaparacho	37°05’06” N 07°47’16” W	210	2	3	—	—	—	—	—	—
*C*. *cardunculus*-2	Morocco: Chefchaouen, Khmis M'Diq	35°04'03”N 5°02'58,0”W	960	1	3	—	—	—	—	—	—
*C*. *humilis*	Spain: Albacete, Riópar	38°25’53” N 02°31’03” W	750	2	2	—	—	—	—	—	—

### DNA extraction, AFLP fingerprinting and plastid DNA sequencing

Total genomic DNA was extracted from 10 mg of silica gel-dried leaf material following the CTAB method of Doyle and Dickson [43] modified by Cullings [44], and with three washing steps with sorbitol buffer following Tel-Zur et al. [45].

The AFLP procedure followed the protocol of Vos et al. [46] as modified by Vilatersana et al. [47]. About 250 ng of genomic DNA was used for the restriction reaction. Restriction and ligation reactions were carried out separately. The final product from ligation and pre-selective was confirmed on agarose gel (1.2%) and diluted 10 times in ddH_2_O. Initially, selective primers were screened using 25 selective primer combinations. Finally, three primer combinations were chosen for the selective PCR (fluorescent dye Vic): *Eco*RI-AAG/*Mse*I-CAG, *Eco*RI-ACG/*Mse*I-CTG, and *Eco*RI-ACA/*Mse*I-CAA. Fragment electrophoresis was performed at Parque Científico de Madrid (Spain) using an ABI 3730 capillary sequencer (Applied Biosystems, California, USA) including GeneScan 500LIZ (Applied Biosystems) as size standard. To avoid laboratory and scoring errors, individuals from different populations were mixed and a positive control was included in each plate.

Five individuals per population were screened for plastid DNA sequence variation, with two plastid DNA regions being amplified and sequenced: the intergenic spacers *ycf*3-*trn*S^GGA^ and *trn*S^GCU^-*trn*C^GCA^. The spacer *ycf*3-*trn*S^GGA^ was amplified with the primers SP43122F and SP44097R as forward and reverse, respectively [48], whereas the spacer *trn*S^GCU^-*trn*C^GCA^ was amplified with the primers *trn*S^GCU^ [49] and *trn*C^GCA^ [50] as forward and reverse, respectively. The profile used for PCR amplification was described by Vilatersana et al. [51] for the *ycf*3-*trn*S region and by Kim et al. [52] for the *trn*S^GCU^-*trn*C^GCA^ region. All reactions were performed in a 25 μL volume following Barres et al. [23]. PCR products were purified with ExoSAP-IT (USB Corp., Cleveland, Ohio, USA), and the amplified DNA segments were sequenced using BigDye Terminator Cycle Sequencing v.3.1 (Applied Biosystems) following the manufacturer’s protocol at the University of Florida ICBR Core Facility on an ABI 3730xl capillary sequencer (Applied Biosystems). Moreover, individuals with rare haplotypes were sequenced twice to avoid contaminations or PCR errors. GenBank accession numbers for all sequences are provided in S1 Table.

#### AFLP data analysis

Electropherograms were analyzed with GeneMarker v.1.85 (Softgenetics, Pennsylvania, USA). Data were scored manually, and the results were exported as a presence/absence (1/0) matrix. The error rate [53] was calculated as the ratio of mismatches (scoring 1 versus 0) over phenotypic comparisons in AFLP profiles with 11.60% of the samples. AFLP loci, when ambiguous or non-reproducible were excluded from the data set. Two of the main types of non-reproducible loci are sequencing artifacts–peaks bell-like–and peaks with a mismatch higher than rate error. The first ones were directly rejected and the last ones were checked twice before accept or reject them.

The genetic relationships among populations were studied using two different methods. For the first, SplitsTree4 v.4.14.3 [54] was used with uncorrected *p* genetic distances and the Neighbor-Net (NN) algorithm to generate a network. The split support of the NN tree was assessed by a bootstrap analysis (1000 replicates) using the same software. For the second, a Principal Coordinate Analysis (PCoA) at population level was constructed based on Nei’s genetic distance using GenAlEx v.6.5 [55].

Three approaches were used to unravel population genetic structure: (1) the Bayesian approach implemented in Structure v.2.3.4 [56]; (2) the Bayesian clustering method implemented in BAPS v.6.0 [57]; and (3) analysis of molecular variance (AMOVA [58]) implemented in Arlequin v.3.5.1.2 [59]. Structure analysis was carried out with 10 independent runs per each *K* value (*K* = 1–15), with a 125,000 burn-in period and 10^6^ Markov chain Monte Carlo iterations. These analyses were performed using a model with admixture, correlated allele frequencies and with no a priori information on the sample location of individuals. The Δ*K* approach [60] was used to identify the most probable number of genetic groups using Structure Harvester v.0.6.93 [61]. The results from different runs were summarized with CLUMPP v.1.1.2b [62]. Graphic visualization of Structure results was represented using Distruct v.1.1 [63]. For BAPS, a spatial clustering algorithm and a mixture analysis of individuals without geographic information was chosen, with 10 replicates from *K* = 2 to *K* = 15 ran. The most likely *K* value was selected according to the highest log marginal likelihood values. The geographic structure of the genetic variability was surveyed by AMOVA. Significance levels of variance components (*F*_ST_ values) were obtained by non-parametric permutations using 10,100 replicates.

Genetic barriers among populations were identified using Barrier v.2.2 software [64], based on Monmonier’s algorithm. Significance of barriers was tested by means of 1000 bootstrapped distance matrices constructed based on Nei’s genetic distance using AFLP-SURV v.1.0 [65]. The relationship between the genetic differentiation (*F*_ST_; estimated through AFLP-SURV) and the geographic distance per population pairs was determined through Mantel tests using 1000 permutations with the Isolation by Distance Web (IBDWS v.3.23 [66]).

Genetic diversity (proportion on polymorphic markers—*PLP*—and the estimates of gene diversity—*H*j—based on Nei’s genetic distance) was measured using AFLP-SURV within each population, within each subspecies and for the whole species, using the approach of Lynch and Milligan [67]. Because differences in sampling intensity between populations could bias the comparisons of genetic diversity, we computed the band richness (*B*r) standardized to the minimal population size in this study (*n* = 5), using a rarefaction method using AFLP-DIV v.1.0 [68]. Multilocus linkage disequilibrium was inferred using the index of association modified to remove the dependency of sample size (*r*_d_), using the program MultiLocus v.1.3 [69]; significance tests were carried out by randomization procedures (1000 times), comparing the observed value with the null hypothesis of no linkage disequilibrium. With the same software, we also tested whether the number of loci sampled was sufficient to detect all genotypic diversity choosing the option “plot genetic diversity vs. number of loci”.

The analyses of genetic structure (Structure, BAPS and AMOVA analyses), Mantel test and genetic diversity parameters were carried out using three datasets: (1) including all populations; (2) including only *C*. *baetica* subsp. *baetica* populations; and (3) including only *C*. *baetica* subsp. *maroccana* populations.

#### Plastid DNA analyses

A statistical parsimony haplotype network was constructed using TCS v.1.21 [70]. The completeness of haplotype sampling across the range of *C*. *baetica* was estimated using the Stirling probability distribution [71], which provides a way to test the assumption that all haplotypes have been sampled [72].

We also evaluated the existence of geographical structure using the “spatial clustering of groups” option implemented in BAPS running ten replicates from *K* = 2 to *K* = 15. The most likely *K* value was chosen according to the highest log marginal likelihood values. Two additional analyses were carried out under the same conditions as AFLP analyses: (1) the evaluation of genetic variability at several hierarchical levels, by means of AMOVA and carried out with Arlequin software, and (2) the identification of genetic barriers among populations using Barrier software (and with Nei’s distance matrix obtained with GenAlEx).

### Ecological niche modeling (ENM)

In order to evaluate the potential distribution of *C*. *baetica* under present climatic conditions and to project it to the past and to the future, ecological niche modeling (ENM) was performed using the maximum entropy algorithm implemented in MaxEnt v.3.3.3k [73]. Information regarding the current distribution of species in their native areas was obtained from presence records included in the Global Biodiversity Information Facility (http://www.gbif.org; only those including pictures were considered), from personal communications (by botanists with expertise in the genus), from Wiklund [31], from the sampling sites of this study, and from the herbarium specimens studied (see below). In total, after removing duplicate records within each pixel [30 arc-sec (ca. 1 km)], 68 presence records of *C*. *baetica* were obtained (47 for subsp. *baetica* and 21 for subsp. *maroccana*). A set of 19 bioclimatic variables and an altitude layer at spatial resolution of 30 arc-sec for the Iberian Peninsula and Morocco under current conditions were downloaded from the WorldClim website (http://www.worldclim.org). After a combination of (1) a correlation analysis in a random sample of 1000 points within the study area (to estimate those variables that were intercorrelated) plus (2) jackknife and percent contribution analyses to evaluate the relative importance of each variable, ten relatively uncorrelated (*r* < |0.85|) variables were selected [bio1 (annual mean temperature); bio2 (mean diurnal range); bio3 (isothermality); bio5 (maximum temperature of the warmest month); bio8 (mean temperature of the wettest quarter); bio9 (mean temperature of the driest quarter); bio15 (precipitation seasonality); bio18 (precipitation of the warmest quarter); and bio19 (precipitation of the coldest quarter) and altitude]. Given that *C*. *baetica* is a calcicolous species, one more variable was added, namely soil pH and provided by ISRIC (World Soil Information; www.isric.org). These 11 variables were used as predictors to calibrate the distribution model in MaxEnt. In the occurrence dataset, we included all reliable point localities, which were randomly split into training data (80%) and test data (20%). Fifty subsample replicates were performed with a default prevalence value of 0.2 and the threshold obtained under the maximum training sensitivity plus specificity rule.

The distribution model under current conditions was projected to the Last Glacial Maximum (LGM; ca. 21 kyr BP) and to the future (year 2070), with the same MaxEnt settings as for the present time. For the LGM, we used paleoclimatic layers simulated under two general atmospheric circulation models: the Community Climate System Model version 4 (CCSM4 [74]) and the Model for Interdisciplinary Research on Climate Earth System Model (MIROC-ESM [75]). For the year 2070, we used the NOAA Geophysical Fluid Dynamics Laboratory Coupled Model 3 (GFDL-CM3 [76]) and the New Earth System Model of the Max Planck Institute for Meteorology (MPI-ESM-LR: http://www.mpimet.mpg.de/en/science/models/mpi-esm/). According to McSweeney et al. [77], these two models are among the best suited for the region of study. These two future projections were run in two different representative concentration pathways (RCPs) that were used in the Fifth Assessment IPCC report, RCP 2.6 and RCP 8.5 [78]. RCP 2.6 represents the most ‘benign’ scenario (i.e., a likely increase of 0.3–1.7°C for *ca*. 2081–2100), whereas RCP 8.5 is the most extreme scenario (a likely increase of 2.6–4.8°C for ca. 2061–2080). Because no scenarios are available for the LGM performance of non-climatic variables (i.e., pH and altitude), but they are probably to be very different between LGM and present time, they were discarded. In contrast, pH and altitude were assumed constant for the 2070 projections, because they will certainly not suffer appreciable changes in just 52 years. The present time and future models were produced using the three same different datasets as in genetic analyses. All ENM predictions were visualized in ArcGIS v.10.2 (ESRI, Redlands, CA, USA), with the aid of Hawth’s Analysis Tools [79]. Thus, in total, 17 models were created.

Niche similarity between the two subspecies of *C*. *baetica* was measured by estimating Hellinger-derived *I* and Schoener's *D* indices [80]; these metrics were calculated with the niche overlap test implemented in the software ENMTools v.1.4.3 [81] using layers generated from MaxEnt. These two indices quantify niche overlap, and range between 0 (ecological niches do not overlap) and 1 (habitats are estimated to be equally suitable for both species). A niche identity test and a background test, with 100 pseudo-replicates each one, were calculated to generate a distribution of the expected values of each index. The identity test generates expected *I* and *D* values from pooling the occurrence points of two compared entities and randomly splitting them into two new groups, while background test uses a buffer zone (10 km) around the occurrence points of each taxa to determine whether ENMs are more (or less) similar than expected by chance. Histograms for each pairwise comparison were constructed after performing both tests to visualize the niche differentiation assuming that empirical values significantly lower than those expected correspond to divergence, whereas those greater than expected correspond to conservatism.

### Morphological analysis

In order to determine the morphological distinctiveness of the two subspecies of *C*. *baetica*, 72 herbarium specimens covering the entire known distribution area (53 from the Iberian Peninsula and 19 from Morocco) were evaluated using two characters recognized as taxonomically relevant by Wiklund [28] (length of the protrusion and length of the spines of the involucral bracts) and two others observed in the present study that we consider that are relevant (relationship between bracteal width and length, and leaf spine length). Florets color and involucral bracts color, two characters defined as taxonomically relevant in the study carried out by Wiklund [28], were not used because some specimens are very old (collected in the first half of 20^th^ century) and these characters could not be determined with exactitude. The four selected characters were measured for all specimens, with those specimens having one or more characters not readily visible being discarded. As there are described hybrids between *C*. *baetica* subsp. *maroccana* and *C*. *humilis* (relatively easily to identify given their intermediate characters [31]), all samples were carefully checked before their inclusion in the present study in order to discard possible hybrids. A complete list of the examined specimens is available in S1 File. The characters were observed using a Zeiss Stemi SV8 binocular stereoscopic microscope or via www.plants.jstor.org (in BM and G specimens), measuring a maximum of five bracts per capitulum and five spines of the first leaf under the capitulum per stem. So as to confirm that the studied variables were uncorrelated, a previous correlation analysis of variables was carried out using SPSS v.22.0 (SPSS Inc., Chicago, IL, USA). A relative correlation (*r* = 0.871) was detected between the length of the protrusion and length of the spines of the involucral bracts, but we decided to keep them because both were used in the most recent taxonomic treatment (i.e., Wiklund [28]). To reduce the overall variation of the morphological characters into new uncorrelated components, a Principal Component Analysis (PCA) was performed (also with SPSS), using herbarium specimens as operational taxonomic units. Due to the different taxonomic treatments used by the herbaria over the years, the herbarium specimens were labeled on the scatterplots according to the geographical area in which they were found, thus forming two groups: Iberian Peninsula (that is, *C*. *baetica* subsp *baetica*) and Morocco (i.e., *C*. *baetica* subsp. *maroccana*).

### Nomenclature

The electronic version of this article in Portable Document Format (PDF) in a work with an ISSN or ISBN will represent a published work according to the International Code of Nomenclature for algae, fungi, and plants, and hence the new names contained in the electronic publication of a PLOS article are effectively published under that Code from the electronic edition alone, so there is no longer any need to provide printed copies.

In addition, new names contained in this work have been submitted to IPNI, from where they will be made available to the Global Names Index. The IPNI LSIDs can be resolved and the associated information viewed through any standard web browser by appending the LSID contained in this publication to the prefix http://ipni.org/. The online version of this work is archived and available from the following digital repositories: PubMed Central and LOCKSS.

## Results

### Genetic analyses

#### AFLP analyses

Using three combinations of primers, a total of 211 unambiguous DNA fragments were scored. The reproducibility ranged between 97.17 and 98.26%, with an acceptable error rate of 2.14% [51]. Eight populations showed private markers (between one and five per population) and three populations harbored fixed markers [a single population in the Iberian Peninsula (ES1) with one marker, and two populations in Morocco (MA1 and MA5) with one and two markers, respectively (Table 2)]. At regional level, 20 private and one fixed markers were present in Morocco, whereas 45 private and five fixed markers occurred in the Iberian Peninsula (Table 2).

**Fig 2:**
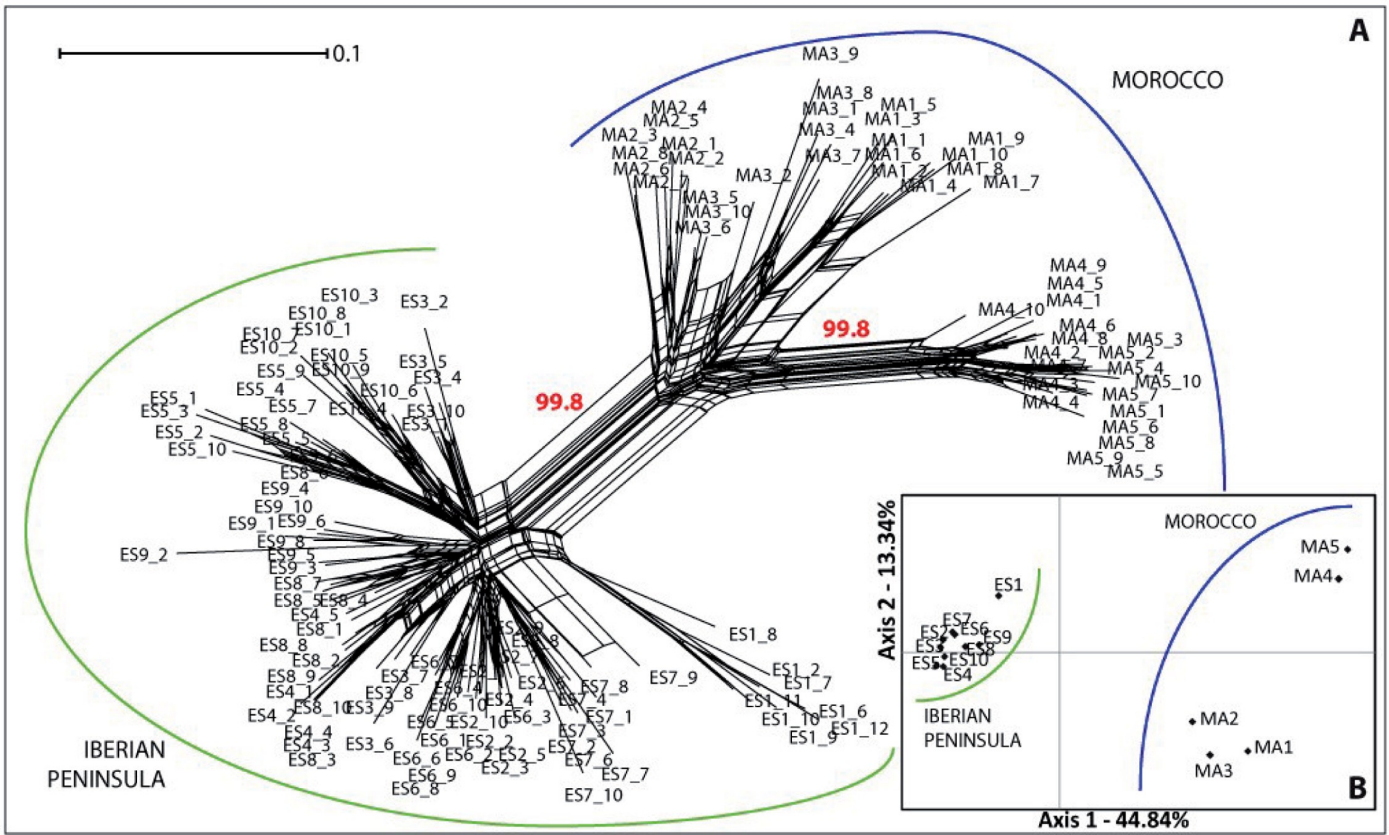
Neighbor-Net and PCoA results based on AFLP studies. (A) Neighbor-Net graph of 135 individuals of *Cynara baetica* based on 211 AFLP loci computed with Splitstree4. In red, bootstrap values. (B) Principal Coordinate Analysis (PCoA) at the population level, based on Nei’s genetic distance among the studied populations of *C*. *baetica*.

The NN diagram showed a genetic split between the populations located at each side of SG (Fig 2A), with high bootstrap support (99.8%). In the NN diagram, two different and well supported (99.8%) subsplits were detected in Moroccan populations. The first includes Rif and northern Atlas populations (MA1–MA3), and the other, southern Atlas populations (MA4 and MA5). Identical results were shown by the PCoA analysis, whose first two components explained 57.18% of the total genetic variance (Fig 2B).

**Fig 3:**
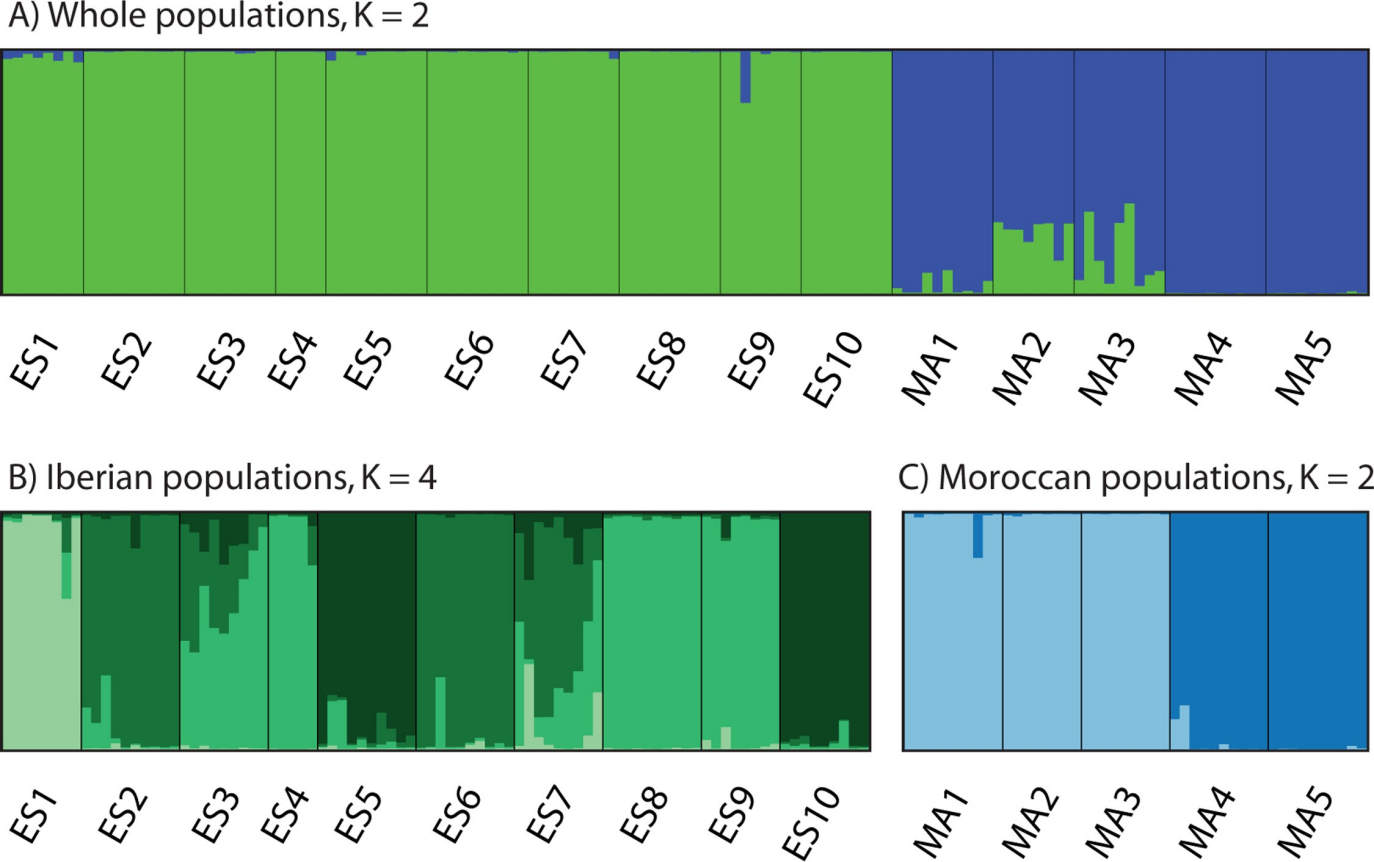
The most optimal *K* results using Bayesian clustering analyses with Structure based on AFLP results. (A) Using whole populations of *Cynara baetica* (*K* = 2). (B) Using only Iberian populations of *C*. *baetica* (*K* = 4). (C) Using only Moroccan populations of *C*. *baetica* (*K* = 2).

**Fig 4:**
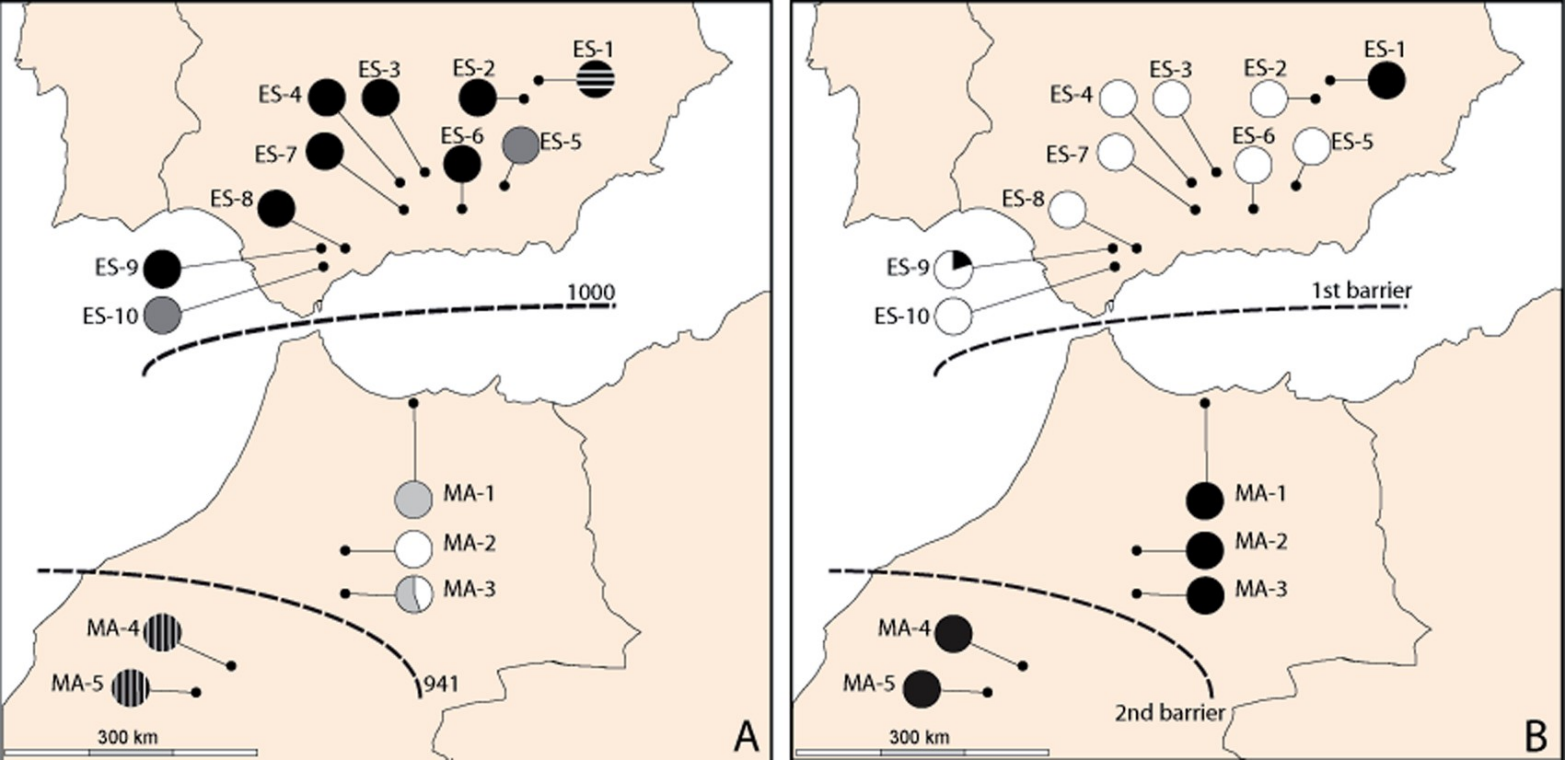
Geographical distribution of the best K value inferred by the software BAPS and barriers to gene flow. (A) *K* = 6 for AFLP results. (B) *K* = 2 for plastid DNA dataset. Dotted lines represent the barriers to gene flow detected by the Barrier software. Barriers for AFLP data are based on 1000 bootstrapped *F*_ST_ matrices.

The clusters defined by the Bayesian Structure analysis with all dataset corroborated NN splits structure, as Δ*K* indicated two optimal genetic groups (*K* = 2; S1 Fig), one grouping the Moroccan populations and the other corresponding to the Iberian populations (Fig 3A). When Structure was carried out selecting only the Iberian populations, four optimal genetic groups were detected (*K* = 4; Figs 3B and S1) with high levels of admixture in some populations (ES3 and ES7). Only two genetic groups (*K* = 2; S1 Fig) were detected when Structure was carried out for the Moroccan populations alone, with almost no admixture between them (Fig 3C). BAPS analysis identified *K* = 6 as the optimal number of genetic groups (Fig 4A), three on each side of SG, which is roughly consistent with the pattern shown by the NN diagram (Fig 2A); populations ES1, ES5 + ES10, MA4 + MA5 had their own groups in both cases. When BAPS was carried out for each subspecies, the results were inconsistent (data not shown). The Mantel test showed a highly significant positive correlation between the pairwise genetic differentiation (*F*_ST_) and the geographic distance for the whole dataset (*r* = 0.706; *P* < 0.005) and for the Moroccan populations (*r* = 0.698; *P* < 0.005), but not for the Iberian populations (*r* = 0.002; *P* < 0.005). Barrier analysis also revealed a major boundary separating both sides of the SG, and a secondary boundary separating the two southern populations (MA4 and MA5) from the northern Moroccan populations (MA1–MA3; Fig 4A).

With no regional grouping, AMOVA attributed most of the genetic variation to among populations (65.60%; Table 3). When the among-regions component was added (Iberian Peninsula versus Morocco), 42% of the total variation was among groups, 31% to among populations within groups and 27% to within populations. However, when each region was explored separately, the among-populations component was considerably higher (48.39% in Morocco and 61.47% in the Iberian Peninsula).

**Table 3:** Results of analysis of molecular variance (AMOVA). AMOVA results for AFLP phenotypes and plastid DNA haplotypes from populations of *Cynara baetica*. (* = *p* < 0.05 after 10,100 permutations).

	AFLP					plastid DNA				
	d.f.	Σ squares	Var. comp.	% variation	*F*_ST_	d.f.	Σ squares	Var. comp.	% variation	*F*_ST_
**Whole dataset**					0.656					0.924
Among populations	14	1991.330	14.955	65.60*		14	34.427	0.484	92.36*	
Within populations	120	941.056	7.842	34.40*		60	2.400	0.040	7.64*	
**Nested analyses**										
***Iberian Pen*.*—Morocco***					0.729					0.950
Among groups	1	832.108	12.071	41.64*		1	19.107	0.538	66.77*	
Among populations within groups	13	1159.222	9.076	31.31*		13	15.320	0.228	28.27*	
Within populations	120	941.056	7.842	27.05*		60	2.400	0.040	4.97*	
**Iberian populations**					0.484					0.830
Among populations	9	648.246	7.320	48.39*		9	9.160	0.196	83.02*	
Within populations	78	609.094	7.809	51.61*		40	1.600	0.040	16.98*	
**Moroccan populations**					0.618					0.882
Among populations	4	510.975	12.772	61.77*		4	6.160	0.300	88.24*	
Within populations	42	331.961	7.904	38.23*		20	0.800	0.040	11.76*	

The percentage of polymorphic bands (*PLP*) ranged from 68.2% (ES7) to 12.3% (ES4), gene diversity (*H*j) from 0.142 (ES7) to 0.058 (MA4), and band richness after rarefaction [*B*r_(5)_], from 1.245 (ES5) to 1.128 (ES4) (Table 2). The *PLP* and *H*j of Moroccan populations (67.3% and 0.195, respectively) were higher than those of Iberian ones (43.6% and 0.163, respectively). All populations showed some degree of linkage disequilibrium (*r*_d_) except for ES6 (Table 2). MultiLocus analysis also revealed that scoring a higher number of loci did not increase the genotypic diversity.

#### Plastid DNA analyses

The combined two plastid DNA regions corresponded to 1365 bp (*ycf*3-*trn*S, 790 bp; *trn*S^GCU^-*trn*C^GCA^, 575 bp) and yielded 6 haplotypes: three in the Iberian Peninsula (haplotypes 1, 2, and 3) and three in Morocco (haplotypes 4, 5, and 6). Two of these haplotypes (haplotypes 2 and 3) were shared with the outgroups (Table 2). All populations harbored only one haplotype, with the exception of ES9 and MA2, each having two haplotypes each (Fig 1 and Table 2). The most widely distributed haplotype was haplotype 1, present in 58.67% of all studied individuals. The haplotype network showed that the maximum number of mutational steps between different haplotypes in *C*. *baetica* is four, whereas that between *C*. *baetica* and outgroups is five, indicating low genetic differentiation between the haplotypes and also between the three different outgroups used. The completeness of haplotype sampling estimated using Dixon’s [72] method was 0.99 (the most likely value of haplotypes = 6), suggesting that all haplotypes present in the species had been sampled.

BAPS analysis revealed two genetically distinct groups (Fig 4B). The first of these groups was characterized by individuals harboring haplotype 1, the most common one, whereas the second group included the remaining individuals. Barrier software identified the same barriers as for the AFLP dataset (Fig 4B).

The AMOVA attributed practically all the genetic variation to the among-populations component (92.36%; Table 3). As for the AFLP, when populations were divided into the two groups separated by the SG (Iberian Peninsula and Morocco), most (67%) of the total variation was among groups (with 31% among populations within groups and 29% within populations). When the AMOVA was computed separately by regions, however, a very large part of the variation was due to differences among populations (83% in Morocco and 88% in the Iberian Peninsula).

### Ecological niche modeling

**Fig 5:**
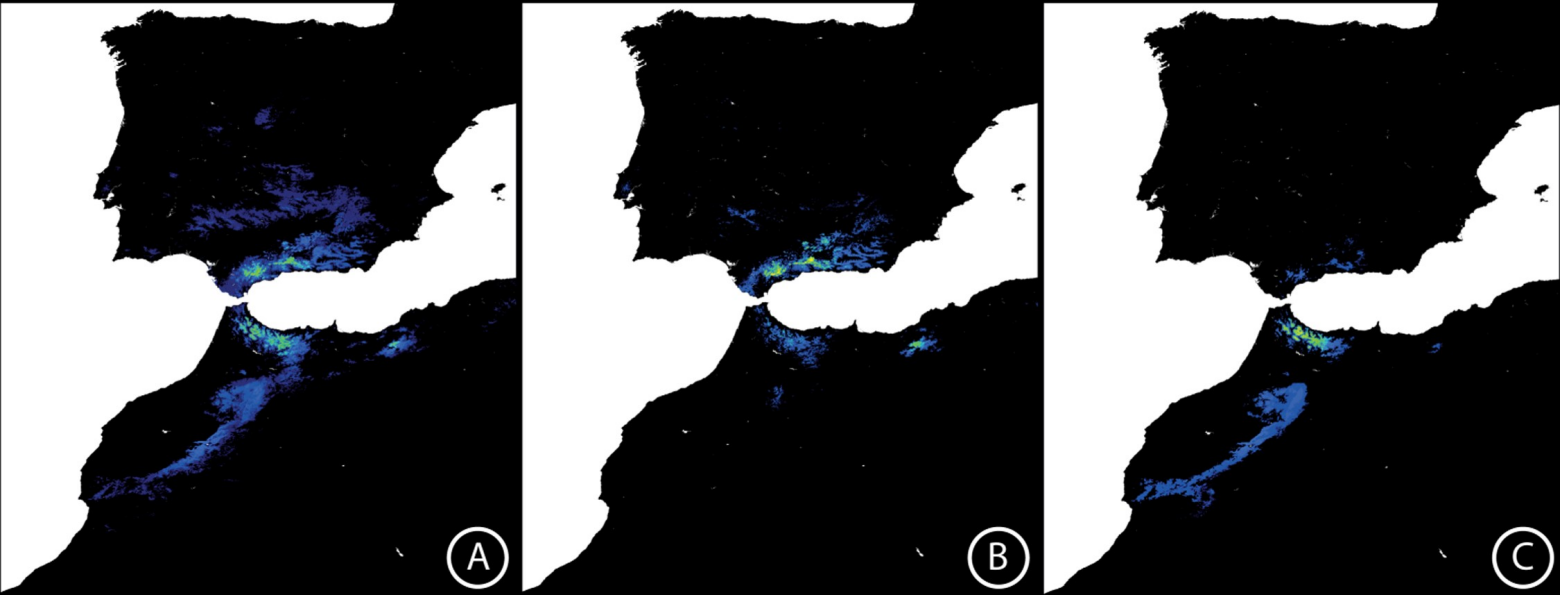
MaxEnt distribution map showing the environmental suitabilty for *Cynara baetica* for the present time. (A) *C*. *baetica* s.l., (B) *C*. *baetica* subsp. *baetica*, and (C) *C*. *baetica* subsp. *maroccana* (C). Warmer colors depict higher environmental suitability.

All ENM for the present time generated with MaxEnt (Fig 5) had an area under the curve (AUC) > 0.97, indicating that the models perform well at predicting species distribution (Table 4). According to jackknife tests, pH, precipitation of warmest quarter (bio18) and precipitation of the coldest quarter (bio19) were the most informative climatic variables for predicting the niche of *C*. *baetica* at specific level and of *C*. *baetica* subsp. *baetica* (Table 4). By contrast, bio19, altitude and bio18 were the three most important variables for predicting the niche occupied by *C*. *baetica* subsp. *maroccana* (Table 4).

**Table 4:** Predicted potential distribution of *Cynara baetica*, with model performance details. The three most important variables for each model are included.

Model	AUC±sd	Maximum training sensitivity plus specificity logistic threshold	Total predicted area (km^2^)	Difference respect to present (km^2^ and %)	Overlap with present (km^2^ and %)	Main variables
**Present**						
*Cynara baetica*	0.977 ± 0.006	0.0329	107,963	—-	—-	pH > bio18 ≈ bio19
*C*. *baetica* subsp. *baetica*	0.980 ± 0.010	0.0246	31,726	—-	—-	pH > bio18 > bio19
*C*. *baetica* subsp. *maroccana*	0.986 ± 0.009	0.0784	35,826	—-	—-	bio19 > altitude ≈ bio18
**LGM-CCSM**	0.944 ± 0.032	0.0619	153,928	45,965 (42.57%)	40,390 (37.41%)	bio18 > bio19 > bio8
**LGM-MIROC**	0.956 ± 0.030	0.0582	335,398	227,435 (210.66%)	72,163 (66.84%)	bio18 > bio19 > bio8
***Average LGM***	—-	—-	*244*,*663*	*136*,*700 (126*.*62%)*	*56*,*276 (52*.*13%)*	—-
**2070-GFDL-2.6**						
*Cynara baetica*	0.965 ± 0.018	0.0253	50,337	-57,626 (-53.38%)	45,354 (42.01%)	pH > bio18 ≈ bio19
*C*. *baetica* subsp. *baetica*	0.973 ± 0.018	0.0214	14,802	-16,924 (-53.34%)	10,473 (33.01%)	pH > bio18 > bio19
*C*. *baetica* subsp. *maroccana*	0.974 ± 0.036	0.0772	6,519	-29,307 (-81.80%)	5,877 (16.40%)	bio19 > bio18 > altitude
**2070-GFDL-8.5**						
*Cynara baetica*	0.968 ± 0.019	0.0230	21,059	-86,904 (-80.49%)	11,668 (10.81%)	pH > bio18 ≈ bio19
*C*. *baetica* subsp. *baetica*	0.977 ± 0.014	0.0256	1,284	-30,442 (-95.95%)	1,207 (3.80%)	pH > bio18 > bio19
*C*. *baetica* subsp. *maroccana*	0.968 ± 0.041	0.0589	3,102	-32,724 (-91.34%)	1,958 (5.47%)	bio19 > bio18 > altitude
**2070-MPI-2.6**						
*Cynara baetica*	0.959 ± 0.025	0.0272	49,970	-57,993 (-53.72%)	48,964 (45.35%)	pH > bio18 ≈ bio19
*C*. *baetica* subsp. *baetica*	0.970 ± 0.017	0.0374	25,879	-5,937 (-18.71%)	24,205 (76.29%)	pH > bio18 > bio19
*C*. *baetica* subsp. *maroccana*	0.966 ± 0.041	0.0355	30,063	-5,763 (-16.09%)	22,809 (63.67%)	bio19 > bio18 > altitude
**2070-MPI-8.5**						
*Cynara baetica*	0.962 ± 0.018	0.0224	18,612	-89,801 (-83.18%)	15,252 (14.13%)	pH > bio18 ≈ bio19
*C*. *baetica* subsp. *baetica*	0.972 ± 0.016	0.0215	14,937	-16,789 (-52.92%)	11,835 (37.30%)	pH > bio18 > bio19
*C*. *baetica* subsp. *maroccana*	0.972 ± 0.039	0.0718	10,295	-25,531 (-71.26%)	7,854 (21.92%)	bio19 > bio18 > altitude
***Average 2070***						
*Cynara baetica*	—-	—-	*34*,*995*	*-72*,*968 (-67*.*59%)*	*30*,*310 (28*.*07%)*	—-
*C*. *baetica* subsp. *baetica*	—-	—-	*14*,*226*	*-17*,*500 (-55*.*16%)*	*11*,*930 (37*.*60%)*	—-
*C*. *baetica* subsp. *maroccana*	—-	—-	*12*,*495*	*-23*,*331 (-65*.*12%)*	*9*,*625 (52*.*13%)*	—-

**Fig 6:**
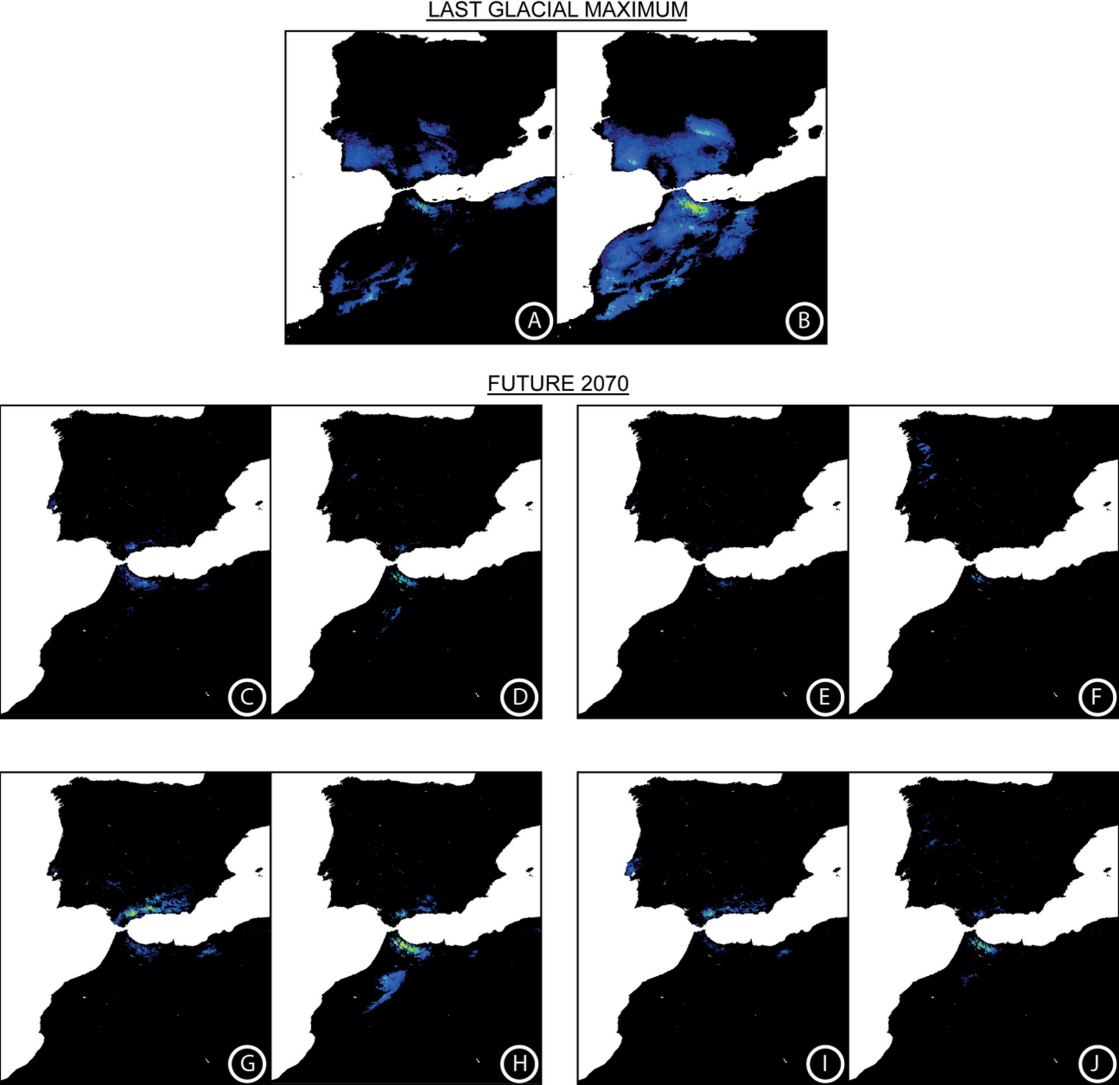
MaxEnt distribution map showing the environmental suitability (A–J) for *Cynara baetica* at the Last Glacial Maximum (LGM) and under different future conditions. LGM according to (A) CCSM and (B) MIROC models. Expected environmental suitability for the year 2070 under different conditions: (C) *C*. *baetica* subsp. *baetica* under GFDL-CM3 model with RCP 2.6; (D) *C*. *baetica* subsp. *maroccana* under GFDL-CM3 model with RCP 2.6; (E) *C*. *baetica* subsp. *baetica* under GFDL-CM3 model with RCP 8.5; (F) *C*. *baetica* subsp. *maroccana* under GFDL-CM3 model with RCP 8.5; (G) *C*. *baetica* subsp. *baetica* under MPI-ESM-LR model with RCP 2.6; (H) *C*. *baetica* subsp. *maroccana* under MPI-ESM-LR model with RCP 2.6; (I) *C*. *baetica* subsp. *baetica* under MPI-ESM-LR model with RCP 8.5; and (J) *C*. *baetica* subsp. *maroccana* under MPI-ESM-LR model with RCP 8.5. Warmer colors depict higher environmental suitability.

Regarding the LGM scenario, AUC values were reasonably high (AUC > 0.94; Table 4), the most important variables being bio18, bio19, and mean temperature of the wettest quarter (bio8). The two LGM models suggested a decrease in the present distribution area of *C*. *baetica* compared with the reconstructed LGM palaeodistribution, although differing its extent, whereas MIROC model predicted an LGM area three times the size of the current one. With the CCSM model, the LGM palaeodistribution only exceeds ca. 40% the current area; Fig 6 and Table 4).

A high level of predictive performance was obtained for the future distribution of the species (AUC > 0.95; Table 4), the same variables as those for the present being the most important in all cases. Contrary to the LGM, the suitable area for the year 2070 was greatly reduced (on average, by more than 67% when all populations were included, and by 55–65% when populations from both sides of SG were studied separately; Fig 6 and Table 4).

**Fig 7:**
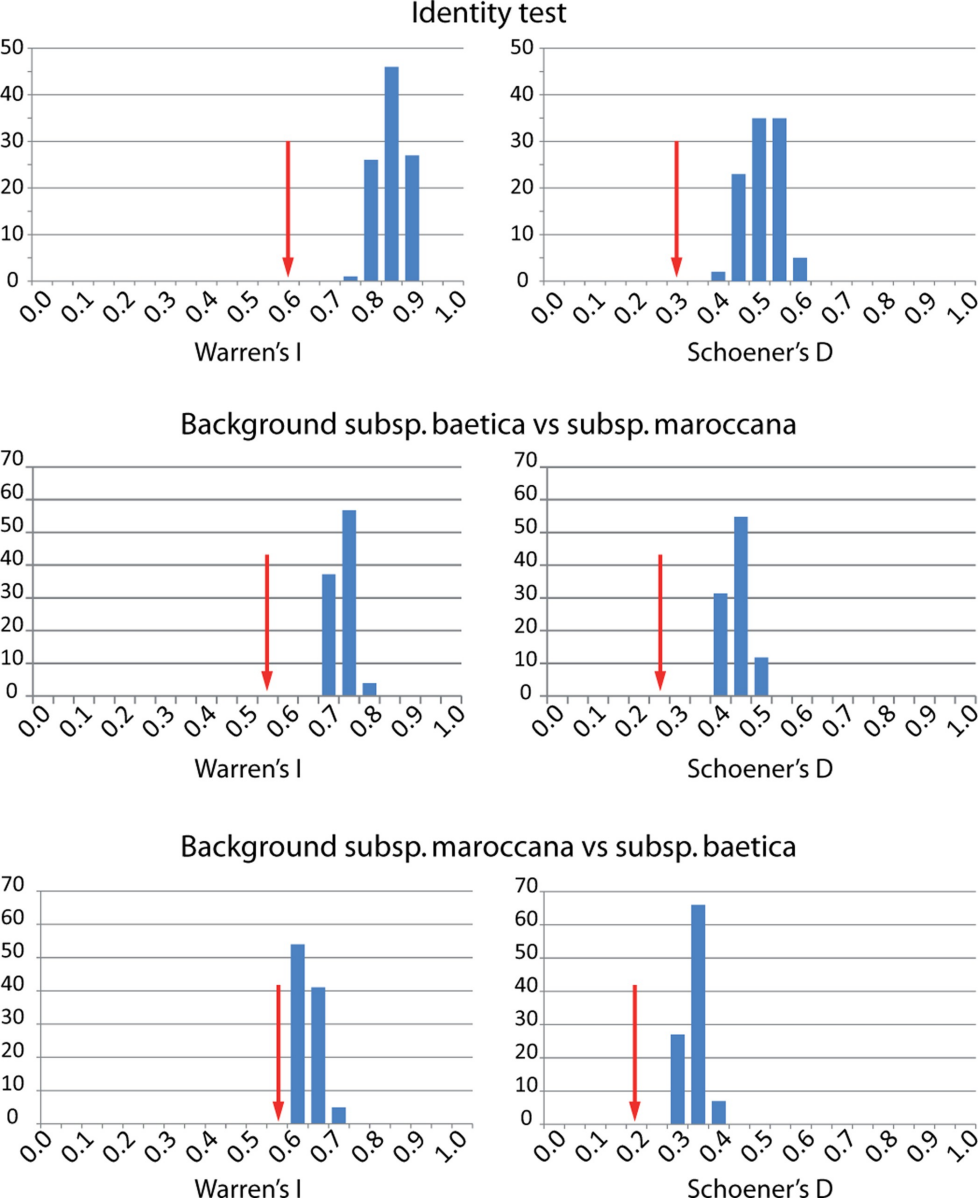
Observed niche overlap values for Hellinger-derived *I* and Schoener’s *D* indices compared with a null distribution. In all cases, the similarity score (red arrow) is less than that predicted by the null hypothesis for niche equivalency, indicating that the environmental niches are not equivalent.

Regarding the niche similarity analyses, the niche identity test and the background test revealed that both *I* and *D* values for the null distribution were significantly greater (*P* < 0.01) than those observed ones (Fig 7); thus, this clearly indicates niche differentiation between both subspecies of *C*. *baetica*.

### Morphological analyses

**Fig 8:**
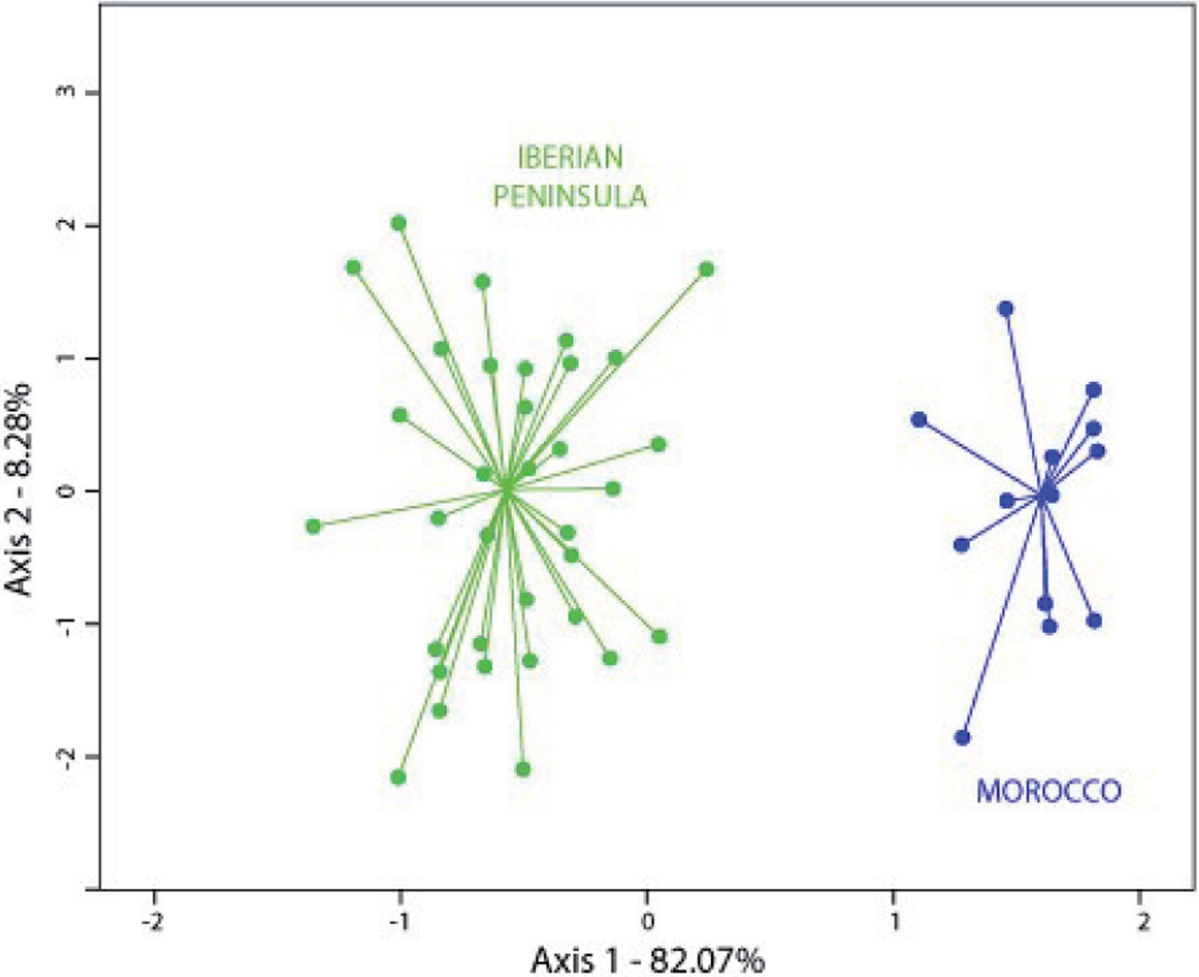
Scatterplot of the first two axes from the Principal Component Analysis (PCA) for the 72 individuals studied of *Cynara baetica*. Fifty-three individuals from the Iberian Peninsula (*C*. *baetica* subsp. *baetica*, in green) and 19 individuals from Morocco (*C*. *baetica* subsp *maroccana*, in blue).

In the PCA (Fig 8), the first axis accounted for 82.1% of the variation whereas the second axis accounted for 8.3%. The two geographical groups (that is, the two subspecies) appear well separated in the scatterplot (Fig 8). For the first axis, the three characters with a highest contribution to the variation (and, thus, contributing most to the differentiation of groups) were length of the protrusion of the involucral bracts, length of the spines of the involucral bracts, and relationship between bracteal width and length. For the second axis, only leaf spine length had a remarkable contribution (data not shown).

## Discussion

### The role of the Strait of Gibraltar in the evolution of Cynara baetica

During the course of time, SG has acted as an effective barrier to gene flow in some taxa, stimulating genetic divergence (e.g., [82,83]). Both nuclear and plastid data analyses clearly indicate the presence of two separate lineages within *C*. *baetica*, each one on either side of SG. Despite the fact that speciation of the genus *Cynara* began ca. 12 Mya [23], only six haplotypes were obtained in this study. Given that chloroplast markers generally require much less time to fix novel mutations compared to nuclear ones [84], these results suggest that separation of these two lineages could have been relatively recent, most likely after the last reopening of SG at late Miocene (5.3 Mya [6]), in a vicariance process similar to that reported for many other animal and plant lineages (e.g., [85,86]). In *C*. *baetica*, such allopatric divergence is supported by the high number of private markers for each of the two lineages (65 out of 211 markers, that is, 30.8%; Table 2) and the genetic structure patterns detected in the nuclear results (Figs 2, 3 and 4). It would therefore seem that the reduction in distance between both sides of SG that occurred at each of the glacial maxima (from the current 15 km to 5 km at the LGM [87]) would not have been sufficient for genetic exchange and for the reversion of this genetic divergence. The low distance dispersal observed for other *Cynara* species (less than 50 m [88]) and the weaker winds for the LGM than for the present in the SG region [89] may also explain how this genetic divergence is maintained.

Such genetic divergence is reflected also in morphological (Fig 8) and ecological niche analyses. The last ones show unequivocal signs of divergence as: (i) the models run with populations from one side of SG only are generally not able to reconstruct the current distribution of the species on the other side of SG (Fig 5A and 5B), (ii) the niches of the two subspecies do not overlap (Fig 7). Indeed, the northern lineage (subsp. *baetica*) occupies more humid niches (precipitation of the warmest quarter = 35.55 mm ± 12.83 vs. 28.33 mm ± 12.49; precipitation of the coldest quarter = 284.00 mm ± 117.67 mm vs. 249.48 ± 68.16), with lower pH (6.71 ± 0.51 vs. 6.98 ± 0.46) and located at lower elevations (909.63 m ± 333.76 vs. 1298.73 m ± 342.74) when compared with the northern lineage (subsp. *maroccana*). The Moroccan lineage, at the same time, can be split into two distinct genetic groups: the northern group, formed by populations MA1–MA3, and the southern group, formed by populations MA4–MA5 (Figs 2, 3C and 4), with a high bootstrap support. The existence of these two Moroccan genetic groups is also suggested by the Barrier software results for both genetic datasets, which indicate the presence of a secondary but significant barrier between High Atlas and Middle Atlas regions (Fig 4). The two genetic Moroccan clusters are, in fact, characterized by a strong genetic singularity; the northern cluster has 41 markers that are not present in the southern cluster. Meanwhile the southern cluster has ten markers that are not present in the northern cluster. In addition, all the haplotypes found in each group are private (Fig 1 and Table 2). The colder and wetter climate of the Late Miocene (11.6–5.3 Ma) could have resulted in favorable conditions for the species, probably allowing continuous gene flow throughout North Africa [90]. However, a period of documented cyclic fluctuations between more humid phases and relatively drier phases in vegetation and climate in north-western Africa that occurred approximately between 3.7 and 1.7 Mya [91,92] would have favored genetic differentiation; such fluctuations became even more evident with the onset of the Pleistocene, with some conspicuous examples such as the periodical ‘greening’ of the Sahara [93]. The pollen record from Lake Ifrah, a mountain lake located on Middle Atlas (33° 33’ N, 04° 56’ W; 1610 m), indicates that the LGM climatic conditions were much colder and drier than at present (between -10°C and -15°C in January instead of the current 5°C and with a precipitation of 200–300 mm compared to the present 900 mm [94,95]). These conditions may have enhanced the isolation of *C*. *baetica* at mountain ranges along the Atlas (where the species may have benefited from the orographic rainfall) and/or at other climatically favorable areas, including gorges, closed valleys and in general, places that were sheltered from the harsh conditions experienced during the cold periods of the Pleistocene [10]. The genetic singularity commented upon above suggest the presence of at least three glacial refugia in Morocco: the first one in the Rif Mountains, the second one in the Middle Atlas and, finally, the third one in the High Atlas. These three putative refugia are also predicted in both LGM scenarios inferred from niche models (Fig 6A and 6B) and, indeed, are in concordance with those proposed by Médail & Diadema [10] for plants in generally found in the Mediterranean Basin.

The topographical complexity of this area, together with the isolated nature of the several sections of the Atlas, generally separated by dry basins and dry plateaux (with annual rainfalls usually below 400 mm), prevented more recent genetic contact between these two clusters. To our knowledge, this pattern of two different clusters separated by the split between High and Medium Atlas is scarcely documented for plants (e.g., *Hypochaeris leontodontoides* Ball [96]), but is more common amongst animals (e.g., the Mediterranean turtle *Mauremys leprosa* [97], or the bat *Myotis nattereri* [98]) and has even been documented for human populations [99]. Several factors may account for the existence of this effective barrier to biota between the Middle and High Atlas, and these may be both climatic and geomorphological in nature: (i) the two ranges are almost totally separated by two dry bassins, the Bahira Tadla Bassin in the east and the Missour Bassin in the west, connected only by a narrow strip of less than 50 km long and having precipitations similar to those of the Middle and High Atlas; (ii) the two ranges are partially separated by the Moulouya River; (iii) the Missour Basin was still being formed during the Pliocene/Pleistocene, whereas the Middle and High Atlas are much older (the most superficial layers are from Middle-Upper Jurassic [100–102]); and (iv) the Middle Atlas basaltic province, the largest and youngest volcanic field in Morocco, with a hundred well-preserved strombolian cones and maars which emitted numerous mafic lava flows that, which is located near Ifrane (33° 15’ N, 05° 10’ W), at the north-western margin of the the Missour Bassin [103]. In addition, the aforementioned <50 km connection between both the Middle and High Atlas was probably subjected to extremely cold and dry LGM climates, and probably covered by ice, judging from the fossil record of Ait Ichou swamp (32°41’ N, 5°33’ W [104]).

Concerning the Iberian Peninsula, the Baetic Ranges are emerging as an extraordinary reservoir of plant genetic diversity, involving both narrowly endemic and geographically widespread taxa [105]. The Iberian population pattern, comprising a high level of admixture (see AFLP results in Table 3), can be explained by a greater habitat continuity in the LGM than at present time (this is particularly evident when Figs 5A and 6A are compared). Indeed, a combination of pollen fossil records and vegetation reconstructions suggest a scenario of more or less continuous steppe or herbaceous vegetation combined with forest patches or open woodlands for the southern Iberian Peninsula (with the only exception being the highest altitude ranges, such as Sierra Nevada) at the LGM; such open vegetation would have provided a suitable habitat for the species in Morocco. Conversely, steppe and open woodlands would have been much less continuous or even absent at this time [106–109]. In the specific case of *C*. *baetica* subsp. *baetica*, however, hybridization could also be responsible of some of the genetic diversity observed in addition to the occurrence of refugia in the Baetic Mountains. First of all, the haplotype 3, that occurs in all studied individuals from the karst range of Calar del Mundo (population ES1, Fig 1B), may have originated here. This haplotype is shared with three different individuals of *Cynara cardunculus* (two from Portugal and the other one from Morocco, Table 2). This might be the result of chloroplast capture, a common feature of this genus [110], and posterior fixation within the population. Population ES1 represents the northernmost distribution of the species and forms a very distinct genetic cluster in all the analyses (Figs 1B, 2A, 3B and 4) probably due to the action of this mountain as refuge for a large number of plant species during the glaciations [111] and, indeed, it is located within one of the over 50 putative plant refugia of the Mediterranean Basin proposed by Médail & Diadéma [10]. That haplotype 3 is limited to ES1 may be simply due to the fact that this population was not a source of recolonization after the LGM; such a phenomenon is well documented for the Mediterranean Basin, where ‘rear edge’ or ‘range margin’ refugia were blocked, and therefore had a limited contribution (if any) to post-glacial recolonization [112–114]. Finally, the existence of haplotype 2 can also be explained by hybridization too, as documented by Robba et al. [30]. This haplotype is shared with *C*. *algarbiensis* and *C*. *humilis* and it is found only in one individual in ES9 (Fig 1), a population where *C*. *baetica* grows alongside *C*. *humilis*. This coexistence between different *Cynara* species is common in the Iberian Peninsula, where an altitudinal gradient is observed, with *C*. *cardunculus* occurring at the foot of the mountain, followed by *C*. *humilis* and finally *C*. *baetica* subsp. *baetica* at the mountaintops [115].

It is well known that long-lived and outcrossing species retain most of their genetic variability within populations [116]. In *C*. *baetica*, however, the within-population diversity levels found in AFLP (Table 3) are very reduced and, therefore, *F*_ST_ values (for non-hierarchical AMOVA, *F*_ST_ = 0.656) are much higher than those expected for short-lived perennial herbs (average *F*_ST_ = 0.41 [116]) and for other Baetic-Rifan perennial herbs (e.g., *Astragalus edulis*, *F*_ST_ = 0.289 [117]; *Carex helodes*, *F*_ST_ = 0.500 [18]). When AMOVA was run separately for both geographic regions (and, therefore, the among-region component is removed) the *F*_ST_ value for the Iberian Peninsula approaches the reference value (*F*_ST_ = 0.484), but is still high in the Moroccan populations (*F*_ST_ = 0.618). Reasons for this may include the low seed dispersal found in the genus *Cynara* [88] and the coexistence of evolutionarily distinct lineages within the pool of studied populations. This last point is particularly applicable to the range of Moroccan species, where two highly divergent genetic clusters were identified. And *F*_ST_ differences between Morocco and the Iberian Peninsula can be explained by the lack of geographical barriers in the latter. Finally, the linkage disequilibrium (*r*_d_) observed in practically all populations (Table 2) can be explained as a biass towards small populations [118,119].

### Taxonomic status of Cynara baetica

There is an increased tendency to use a multidisciplinary approach to study the delimitation of plant species (also known as integrative taxonomy [40]) involving three different tools: statistical analyses of morphological variation, population genetic data; and ecological niche analyses, including both modeling and niche conservatism/divergence tests (e.g., [38]). As shown by our results, niche overlap tests (clearly suggestive of niche divergence among lineages; Fig 7), morphological studies (Fig 8 and the different floret color observed during fieldwork and also in some well-preserved specimens), as well as the neatly-differentiated genetic clusters corresponding to the two subspecies (Figs 2, 3A and Table 3), do not support the current taxonomical treatment of these taxa. Instead, all lines of evidence clearly indicate that *C*. *baetica* should be divided into two different species, returning therefore to the old classification. Here, and according to the International Code of Nomenclature for algae, fungi and plants [120], we propose the following redefinition of the two subspecies proposed by Wiklund [28] as *C*. *baetica* (Spreng.) Pau instead of *C*. *baetica* subsp. *baetica*, and *C*. *hystrix* Ball instead of *C*. *baetica* subsp. *maroccana*.

Boissier [121] described *C*. *alba* (now a synonym of *C*. *baetica*) but this name is incorrect because it does not conform to the nomenclature standards, as explained thoroughly by Pau [122]. For that reason, its correct name is *C*. *baetica* and not *C*. *alba*.

#### Summary of limits and distribution of the species

*Cynara baetica* (Spreng.) Pau
**SYNONYM:** *Cynara alba* Boissier ex de Candolle, Prodomus systematis naturalis regni vegetabilis. 1838; 7: 304.
**SYNONYM:** *Cynara baetica* (Spreng.) Pau subsp. *baetica*. The genus *Cynara* L. (Asteraceae-Cardueae). Bot J Linn Soc. 1992; 109: 75–123.

*Cynara baetica* is characterized by pale green involucral bracts usually with a dark margin that protrudes 14–22 mm and a 2–4 mm long spine; florets are white and the leaf apex has a 5–9 mm long spine. It occurs in the southern Iberian Peninsula between 500 and 1700 m.

*Cynara hystrix* Ball
**SYNONYM:** *Cynara baetica* (Spreng.) Pau subsp. *maroccana* Wikl. The genus *Cynara* L. (Asteraceae-Cardueae). Bot J Linn Soc. 1992; 109: 75–123.

*Cynara hystrix* has involucral bracts protruding 21–31 mm, pale green and tinged with purple, usually with a dark margin and with a 6–9(–11) mm long spine; florets bright lilac, the leaf apex with a 2–6 mm long spine. It grows in central and northern Moroccan mountains, between 900 and 2100 m.

### Conservation guidelines

Correct species delimitation is of paramount importance in conservation biology because the species is usually the minimum unit for legal protection (red lists, red books, and catalogues of protected taxa all generally uses species—or subspecies—as conservation units). Surveying levels and distribution of genetic diversity, nevertheless, play an important role in species conservation, as genetic variability is a prerequisite for evolutionary adaptation and long-term survival of the species [123]. Finally, ENM has also become an important tool in conservation biology, as it is useful in predicting the effects of climate change on species distribution, allowing one to measure the effectiveness of extant protected areas, and to propose the establishment of new ones (e.g., [124]). Based on our results, we can propose the following conservation guidelines: firstly, concerning *C*. *baetica*, the Spanish Red List should be updated accordance to the proposed taxonomic change. According to the last version of the Red List of Spanish Vascular Flora [29], *C*. *baetica* subsp. *baetica* is listed as Vulnerable (VU) by IUCN criteria of B2ab(i,ii,iii,iv,v) [125]. We propose the retention of this category but under the name *C*. *baetica*. The regional red list of Andalusia, where the taxon is also listed as VU under the name *C*. *baetica* subsp. *baetica* [126] should also be changed in the same way. *Cynara hystrix*, is neither listed for, nor protected in Morocco, but is well known to the local population (e.g., [127,128]), and it is used against some digestive diseases [128]. Recently, Fennane [129] has published a draft of the red list for Compositae in Morocco, and *C*. *hystrix* (named as *C*. *baetica*) is listed as Least Concern (LC). Given the current highly threatened status of the species in this country (S. Massó & R. Vilatersana, pers. obs.; see also below), we recommend that the species should be listed—obviously under the name *C*. *hystrix*—as Endangered (EN) following the IUCN criteria A2ac [125], due to estimated population size reduction of ≥ 50% over the last 10 years by non-reversible causes (e.g., loss of its habitat for cultivating *Cannabis sativa* L. [130]).

With regard to in situ and ex situ measures, and following the study of both AFLP and plastid DNA sequence markers, two evolutionary significant units (ESUs [131]) are proposed, one per taxon. In addition, two management units (MUs [132]) can be defined for each ESU based on endemic haplotypes/fragments, as these may represent singular genetic variants that may have evolved separately from each other and, therefore, deserve special conservation efforts. Accordingly, the genetic singularities detected for population ES1—a private haplotype (that suggests ancient plastid capture, as explained above), and its own separate cluster as defined by AFLP markers (Figs 2, 3B and 4A)—suggest that this population might constitute one of the MUs for *C*. *baetica*. In addition, ES1 is the population with the largest number of private and fixed markers (Table 2). Fortunately, this population is protected at present, as it is located within the Natural Park of Los Calares del Río Mundo y de la Sima. The second MU defined for the Iberian Peninsula consists of all the remaining populations, and these show a high level of admixture (Figs 2 and 3) and haplotype uniformity (Fig 1A). Of this second MUs, at least three populations are covered by the present network of protected areas (PAs): ES2 (Natural Park of Sierras de Cazorla, Segura y las Villas), ES4 (Natural Park of Sierras Subbéticas), and ES6 (Natural Park of Sierra Nevada). Unfortunately, all future ENM projections predict very high habitat loss for 2070 (Fig 6), including nearly all the current protected areas

In Morocco, two MUs can also be defined based on genetic data: the first of these would include the two southernmost populations (MA4 and MA5, which define a well-supported genetic group in Fig 2A), whereas the other consists of the three northernmost populations (MA1–MA3). Of the studied populations in Morocco, only one occurs within any PA (MA4, in the National Park of Ifrane), although there are some literature citations in northern Morocco located in protected areas (see S1 File), including a few within the National Park of Talassemtane. Unfortunately, these last populations were destroyed by local people for the growing of *Cannabis sativa* [130]. Extinct localities in the Rif Mountains may have played a key role in the past because they were located within a putative past refugium (Fig 6B), whereas currently, and the future predictions, they are located within areas of higher probability of occurrence; Fig 6C, 6D, 6F, 6H and 6J).

## Supporting information

S1 Fig Δ*K* statistic of Evanno et al. [58] used to estimate the most likely *K*. (A) *C*. *baetica* s.l., (B) *C*. *baetica* subsp. *baetica*, and (C) *C*. *baetica* subsp. *maroccana* (C).
(PNG)

S2 Fig Principal Component Analysis (PCA) with the 11 variables used in the ecological niche modeling. (JPG)

S1 Table The GenBank accession numbers of the haplotypes per population obtained in this study. (DOCX)

S1 File List of the herbarium specimens used in the morphological study. (DOCX)
