# Supplementary figures and images for: Reinterpretation of an endangered taxon based on integrative taxonomy: The case of Cynara baetica (Compositae)
Source: PLoS One. 2018 Nov 28;13(11):e0207094. doi: 10.1371/journal.pone.0207094 (PMC6261557; doi:10.1371/journal.pone.0207094)

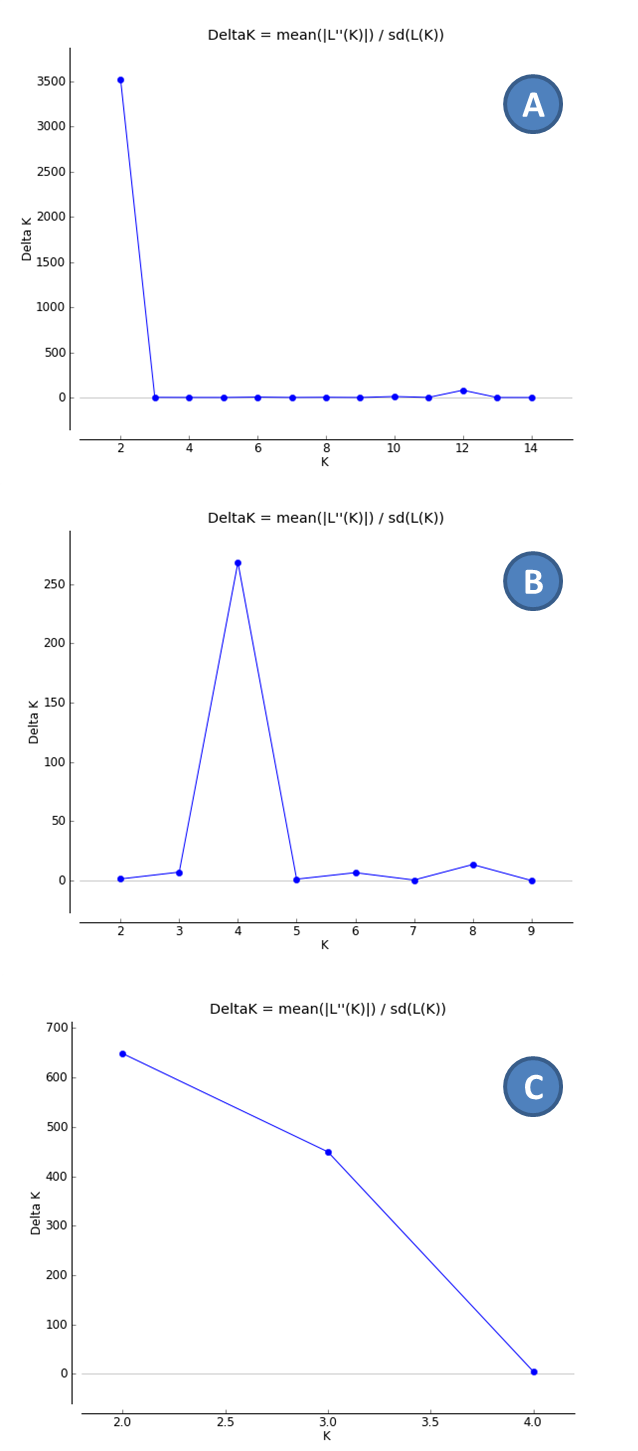

Supplement: S1 Fig — (A) C. baetica s.l., (B) C. baetica subsp. baetica, and (C) C. baetica subsp. maroccana (C). (PNG) [file pone.0207094.s001.png]

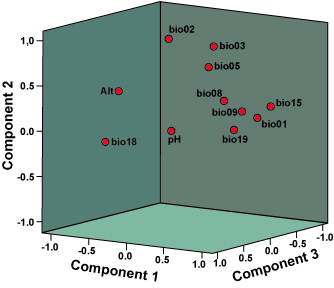

Supplement: S2 Fig — (JPG) [file pone.0207094.s002.jpg]
