# Supplementary material for: Reinterpretation of an endangered taxon based on integrative taxonomy: The case of Cynara baetica (Compositae)
Source: PLoS One. 2018 Nov 28;13(11):e0207094. doi: 10.1371/journal.pone.0207094 (PMC6261557; doi:10.1371/journal.pone.0207094)
Supplement: S1 Table — (DOCX) [file pone.0207094.s003.docx]

**S1 Table. The GenBank accession numbers of the haplotypes per population obtained in this study.**

| Code | Population | Coordinates | Alt. (m) | Hap. | *ycf*3-*trn*S^GGA^ | *trn*S^GCU^-*trn*C^GCA^ |
| --- | --- | --- | --- | --- | --- | --- |
| **IBERIAN PENINSULA** | |  |  |  |  |  |
| ES1 | Spain: Albacete,  Calar del Mundo | 38º29’29” N  02º18’47” W | 1000 | 3 | MH341534 | MH341554 |
| ES2 | Spain: Jaén,  Hornos | 38º13’57’’ N  02º39’48’’ W | 1260 | 1 | MH341535 | MH341555 |
| ES3 | Spain: Jaén,  Locubín | 37º33’58’’ N  03º52’18’’ W | 1100 | 1 | MH341536 | MH341556 |
| ES4 | Spain: Córdoba,  Los Villares | 37º24’08’’ N  04º17’24’’ W | 680 | 1 | MH341537 | MH341557 |
| ES5 | Spain: Granada,  Gor | 37º22’10’’ N  02º58’10’’ W | 1190 | 1 | MH341538 | MH341558 |
| ES6 | Spain: Granada,  El Purche | 37º08’11’’ N  03º28’23’’ W | 1500 | 1 | MH341539 | MH341559 |
| ES7 | Spain: Málaga,  Alfarnate | 36º59’39’’ N  04º15’37’’ W | 1000 | 1 | MH341540 | MH341560 |
| ES8 | Spain: Málaga,  Puerto Martínez | 36º48’13’’ N  04º51’20’’ W | 730 | 1 | MH341541 | MH341561 |
| ES9 | Spain: Cádiz,  Puerto Boyar | 36º45’16’’ N  05º23’39’’ W | 1100 | 1 | MH341542 | MH341562 |
|  |  |  |  | 2 | MH341544 | MH341564 |
| ES10 | Spain: Málaga,  Cortes | 36º37’02’’ N  05º20’32’’ W | 610 | 1 | MH341543 | MH341563 |
| **MOROCCO** |  |  |  |  |  |  |
| MA1 | Morocco: Al Hoceïma,  Bni Hadifa | 35º00’34’’ N  04º11’22’’ W | 1200 | 5 | MH341545 | MH341565 |
| MA2 | Morocco: Ifrane,  Ifrane | 33º30’16’’ N  05º01’24’’ W | 1800 | 5 | MH341546 | MH341566 |
| MA3 | Morocco: Ifrane,  Col du Zad | 33º02’27’’ N  05º03’30’’ W | 2100 | 5 | MH341547 | MH341567 |
|  |  |  |  | 6 | MH341548 | MH341568 |
| MA4 | Morocco: Azilal,  Afourer | 32º10’57’’ N  06º31’16’’ W | 1100 | 4 | MH341549 | MH341569 |
| MA5 | Morocco: Azilal,  Demnate | 31º45’55’’ N  07º00’45’’ W | 900 | 4 | MH341550 | MH341570 |
| **Outgroups** |  |  |  |  |  |  |
| *C.algarbiensis* | Portugal: Algarve, Manchique | 37º18’32’’ N 08º36’27’’ W | 780 | 2 | MH341552 | MH341572 |
| *C. cardunculus*-1 | Portugal: Algarve, Moncaparacho | 37º05’06’’ N 07º47’16’’ W | 210 | 3 | MH341551 | MH341571 |
| *C. cardunculus*-2 | Morocco: Chefchaouen, Khmis M'Diq | 35º04'03’’N 5º02'58,0’’W | 960 | 3 | MH341551 | MH341571 |
| *C. humilis* | Spain: Albacete, Riópar | 38º25’53’’ N 02º31’03’’ W | 750 | 2 | MH341553 | MH341573 |
