# Supplementary material for: Reinterpretation of an endangered taxon based on integrative taxonomy: The case of Cynara baetica (Compositae)
Source: PLoS One. 2018 Nov 28;13(11):e0207094. doi: 10.1371/journal.pone.0207094 (PMC6261557; doi:10.1371/journal.pone.0207094)
Supplement: S1 File — (DOCX) [file pone.0207094.s004.docx]

**S1 File**

List of the herbarium specimens used in the morphological study.

**IBERIAN PENINSULA**

**Albacete:** Riópar, bajo el pueblo viejo, junto a la carretera a Alcaraz, margas triásicas, 30S WH4762, 1000 m. 16-08-2003. *V. J. Arán 5663 & M. J. Tohá* (COA 39861); Dans les champs à Riopar, où il est abondant. Feuilles radicales 10 Juin, fleur 6 Août, 1850. *Bourgeau 733* (G 405626); Villaverde de Guadalimar, valle del arroyo de la Puerta, en la ladera NW del Calar del Mundo, junto a la pista, suelo arcilloso, 30S WH4655, 1100 m. 16-08-2002. *J. Aran 5339 & M. J. Tohá* (VAL 144154);

**Cádiz:** Algar. 09-07-1919. *C. Pau* (BC 33131); Algodonales, Sierra de Líjar. Calizas jurásicas. 500-700 m.s.n.m. 02-08-1980. *A. Aparicio* (MGC 9288); Espera, bordes de la carretera hacia Las Cabezas; a 10 km de Espera. 10-07-2004. *E. López & F. Valtueña* (COFC 29983); Bordes de carretera entre Las Cabezas de San Juan y Espera (CA-4412), a 10 km de Espera. 36.92N 5.88E. 10-07-2004. *E. López & F. J. Valtueña* (UNEX 35497-1); Entre Alcalá de los Gazules y Puerto de Gáliz. Pastizales en los claros del acebuchar-lentisclar. Afloramientos margosos entre areniscas. 10-08-1993. *S. Talavera & B. Valdés* (SEV 211163); Entre Ubrique y El Bosque. 13-07-1978. *J. A. Devesa, J. Rivera & B. Valdés* (SEV 38282); Cruce Grazalema – El Bosque, 30S TF8871, 1190 m. 02-07-1993. *A. Lora & E. Martín-Consuegra* (COA 39865);

**Ciudad Real:** Entre Montiel y Albadalejo, 30S WH17, 980 m, en comunidades de márgenes de carretera, 13-07-1988. *Esteso, Peris & Stübing* (VAL 15390);

**Córdoba:** Priego de Córdoba. El Chaparral, Sierra de Horconera. 14-08-1977. *J. A. Devesa* (SEV 51408); Entre Priego de Córdoba y Carcabuey. 08-1971. *J. Varo* (GDA 2157; GDA 4360).

**Granada:** Cogollos Vega. Prados próximos a Cortijo del Moralejo. 30S VG5529, a 1440 m. 17-08-1988. *O. Socorro, L. Cano, M. L. Arrebola & M. C. Espinar* (GDA 22424); Entre Fuente del Hervidero y Puente de los 7 ojos. 02-08-1984. *C. Quesada & al.* (GDA 20581); Puerto de los Alazores. 31-08-1984. *C. Quesada et al.* (GDA 20593); Puerto de la Inquisición. 22-07-1953. *Rivas & Galians* (SEV 5368); Sierra de Baza. WG03, Gor a 1240 m. Ruderal-viaria. 13-08-1984. *J. Torres, G. Blanca & C. Morales* (GDA 40572); Sierra Nevada, carretera, alt. 1200 m. 17-07-1984. *C. Quesada & P. Sánchez* (GDA 20580); In Sierra Nevada argillosis et supra Alhaurin. 09-1837. *Boissier 124* (G 223658; G 223689; G 223660; G 223661; G 223662; G 223663); Sierra Nevada. 1827. *Willkomm* (G 405623); Regnum Granatense, Sierra Nevada, loc. dicto Purche in campis argillosis. 1500 m. 1879. *Huter, Porta & Rigo* (G 405624);

**Jaén:** La Loma. Villanueva del Arzobispo. Cañada de la Fuensanta. Ruderal. WH02, 800 m. 28-07-199? (SEV 235279);

**Málaga:** Antequera. Sierra de Camarolos. Nacimiento del río Guadalmedina. Entre el Cerro Cruz y los Tajos de Marchena. Pastizales pastoreados sobre suelos con encharcamiento temporal. Arcillas. 30S UF7992. 1350-1380 m. 02-07-2014. *J. García-Sánchez & F. Soriguer* (MGC 79701); Casarabonela. Sierra Prieta. Cañada Real Málaga-Ronda. Camino de Espildora. Arcillas con encharcamiento temporal. 30S UF3374. 650 m. 16-06-2010. *A. V. Pérez Latorre & F. Soriguer* (MGC 71896); Colmenar. Llanos de Marchena. Entre la umbría de Sierra Prieta y la Sierra de Camarolos. Pastizales pastoreados sobre suelos con encharcamiento temporal. Arcillas. 30S UF7991. 1200 m. 15-07-2016. *B. Cabezudo & F. Soriguer* (MGC 83971); Colmenar. Sierra de Camarolos. Llanos de Marchena. Zona hidrófila. Suelo hidromorfo vértico. 30S UF7991. 1200 m. 02-07-2009. *B. Cabezudo, A. V. Pérez Latorre & F. Soriguer* (MGC 70455); Colmenar. Sierra de Camarolos. Nacimiento del río Guadalmedina. Alrededores del puerto en el límite de términos. Pastizales pastoreados sobre suelos con encharcamiento temporal. Arcillas. 30S UF8092. 1300-1350 m. 13-06-2014. *B. Cabezudo & F. Soriguer* (MGC 79782); Cortes de la Frontera. La Sauceda. Pastizal sobre arcillas expansivas. 30S 267478, 560 m. 05-08-2005. *R. Pielfert* (SEV 215142); Entre Cortes de la Frontera y Ubrique. 36.64N 5.45E. 23-07-2003. *J. A. Devesa & T. Rodríguez-Riaño* (UNEX 35499-1); entre Cortes de la Frontera y Ubrique. 22-07-2003. *J. A. Devesa & T. Rodríguez Riaño* (COFC 29984); Gaucín. Sierra del Hacho. Calizas. 30S TF9044. 980 m. 10-08-2008. *O. Gavira* (MGC 68793); Gaucín. El Capitán. Margas y calizas. 30S 0293785 4040729. 160 m. 04-07-2008. *O. Gavira* (MGC 68616); Gaucín. La Gitana. Margas. 30S 0294011 4040346. 200 m. 20-06-2008. *O. Gavira* (MGC 68618); Ronda. Arroyo de los Linarejos. Calizas. 30S UF1163. 860 m. 21-09-1987. *D. Montilla* (MGC 36446); Ronda. Arroyo de los Linarejos. Correolas. 30S UF1162. 860 m. 30-08-1986. *D. Montilla* (MGC 36447); Ronda. Arroyo de los Linarejos. 30S UF1163. 860 m. 21-10-1987. *D. Montilla* (MGC 36468); Ronda, km 126 carretera Alcantara. 22-07-2003. *J. A. Devesa & T. Rodríguez-Riaño* (UNEX 35500-2); Ronda. Pinsapar de la Sierra de las Nieves, 30S UF1867. 03-09-1988. *R. Cáceres & A. Lora* (COA 39830); Ronda, Km-126 de la carretera hacia Alcántara. 22-07-2003. *J. A. Devesa & T. Rodríguez Riaño* (COFC 29985); De Ronda a Coto Rajete. JF06. 27-07-1986. (MGC 18790); Sierra de Ronda, lieux arides, sur le calcaire. 07-08-1889. *Lange* (G 405625; G 405628).

**MOROCCO**

**Al-Hoceïma:** Between Al-Hoceima and Targuist, at 11 km from Targuist. Sandstone. 34.58N, 5.05W. *M. J. Díaz, M. Etlaftski & B. Valdés* (BC 945552); Between Al-Hoceima and Targuist, at 11 km from Targuist. Sandstone. 34.58N 5.05W. 820 m. 03-07-1993. *M. J. Díaz, M. Etlaftski & B. Valdés* (SEV 136264); Targuist, champs, vers 1000 m. 07-07-1931. *Sennen & Mauricio* (BC 139403; BCN 59684; BM 924810; G 23522; VAL 139227); Targuist to Al Hoceima road. 16 km West of Beni-Hadifa. 35.00N, 4.15W. 30S 386413 3871875. Alt 1150 m. Roadside ruderal community. 29-06-1993. *S. L. Jury 11233, L. S. Springate & M. Ait Lafkih* (BC 936821); Hab. inter Targuist et Sok-et-Tnin de Beni Hadifa (Beni Uriaguel), 1200 m, 20-07-1929. *Font i Quer* (G 405620); Hab. Inter Targuist et Sok-el-Tnin de Beni Hadifa (Beni Uriaguel), ad 1200 m. 20-07-1929. *Font i Quer* (BCN 72554);

**Azilal:** En matorral basófilo, unos 18 km al NE de Azilal (Beni-Mellal). 29S QR3653. 1300 m. 12-07-2006. *A. Romo & F. Gómiz* (BC 866385);

**Béni-Mellal:** 1 km below El-Ksiba along road to Kasba Tadla. 29S 777387 360841. Alt. 1000 m. Weed in harvested cereal fields on steep N-facing rocky limestone slope below cliffs. 04-07-1997. *S. L. Jury17428, A. Abaouz, M. Ait Lafkih & A. J. K. Griffith* (MA 616072);

**Chefchaouen:** Alrededores de Tissouka (pueblo). Calizas. 35.08N 5.12W. 1020-1500 m. 24-07-1995. *M. A. Mateos, A. Ortega & F. J. Pina* (SEV 138891). Between the crossing Chaouen-Meknès and Bab-Taza. Damp slime soils. 35.05N 5.14W. 600 m. 29-06-1993. *M. J. Díaz & B. Valdés* (SEV 139116); Beni M’hamed, Oued Anou (transcripción oral). Arroyo y montañas calizas. 35.08N, 5.07W, 1300-1600 m. 28-17-1996. *M. C. García, M. A. Mateos, F. J. Pina & I. Sánchez* (SEV 155349);

**Ifrane:** Ifrane: trockene Wiesen unweit der Station biologique. 1630 m. 02-0-1960. *F. Höpflinger* (G 405621); Ifrane, plateau du n’Treten. 1900 m. 12-07-1937. *J. Gattefossé* (G 405622); Timhadit, steppes arides, 1850 m. 04-08-1924. *E. Jahandiez* (BC 33133).
